# Laboratory-based experimental research into the effect of diagenesis on heated bone: implications and improved tools for the characterisation of ancient fire

**DOI:** 10.1038/s41598-022-21622-5

**Published:** 2022-11-02

**Authors:** Femke H. Reidsma

**Affiliations:** 1 Human Origins Group, Faculty of Archaeology, Leiden University, Leiden, The Netherlands; 2 GeoLab, Faculty of Geosciences, Utrecht University, Utrecht, The Netherlands

**Keywords:** Archaeology, Geochemistry, Characterization and analytical techniques

## Abstract

The use of fire is considered to be one of the most important cultural innovations in human evolution. Understanding the taphonomy of fire remains is an important prerequisite for valid interpretations of hominin fire-related behaviour. Presented here are the results of a series of laboratory-based experiments testing the effect of different pH conditions (acidic, neutral, alkaline) on the physical and chemical properties of heated bone (charred and combusted). By taking a fundamental-research approach the study gives insight into the specific effect of pH exposure and its underlying chemical processes, and provides data that can be applied to heated bone from any context and time period. Results show that diagenesis has a significant impact on the preservation potential of heated bones, as well as on the reliability of the analytical techniques used to reconstruct past heating conditions. The study provides reference data and a toolkit for the analysis of heated bone, that explicitly takes diagenesis into account, and in doing so offers a significant improvement to the accuracy with which we can reconstruct heating conditions and fire-related human behaviour in the past.

## Introduction

The use of fire played a pivotal role in the development of the human niche. Insights into when and where our ancestors first used fire and what they used it for have important implications for our understanding of key aspects of early human lifeways. Recent years have seen increased interest in and discussion of various aspects of ancient fire use, such as the chronology of early fire use (e.g.,^1–4^), the origins of fire production (e.g.,^5,6^), the development of different pyrotechnologies (e.g.,^7–9^), the role of cooking (e.g.,^10^), the costs of fire use (e.g.,^11,12^), the use of fire as a tool for landscape management (e.g.,^13,14^), the use of different fuels (e.g.,^15–17^), the potential of fire as a proxy for the development of cultural transmission and language^18,19^, and various studies focussing on the integrated analysis of hearth features (e.g.,^20–22^).

The archaeological identification of fire use is based on the presence of organic and inorganic thermally altered materials such as charred plant material, bone, ash, lithics, sediments, tar and various (bio)molecules. Patterns of presence and absence of fire proxies inform about the chronology and degree of hominin control of fire, while the properties of the heated materials provide insight into the nature of past fires and their potential functions. Heated bone is one of the most important fire proxies that provides unique information about the nature of ancient fires (i.e., temperature and oxygen availability). Its high information potential is due to the presence of both organic and inorganic components (respectively circa 20 wt% vs 70 wt%, plus an additional 10 wt% water). This composition allows the material to register the effect of both low and high temperatures within a fire as well as variation in oxygen availability. In contrast, charcoal mainly captures low to medium temperatures in the absence of oxygen, because it fully combusts at higher temperatures in the presence of oxygen.

When bone is exposed to heat its organic content is first denatured (i.e., destabilised) and then converted into char and gradually lost. Alongside the decrease in organic content some inorganic compounds are also lost and the bone mineral reorganises into a more crystalline structure. At higher temperatures heated bone is therefore more homogenous and more chemically stable. The difference between bone heated with and without oxygen (i.e., combusted vs. charred bone) mainly relates to: (1) the amount of organics present at a given temperature, and (2) the timing at which changes to organic and inorganic compounds occur. Charred bone has more organics and lower crystallinity, while combusted bone has less organics and higher crystallinity, and the two types become more chemically distinct with increasing temperature (for details see^23,24^). Within this study ‘charred bone’ refers to the full temperature range of bone heated under reducing conditions, while ‘combusted bone’ refers to the full temperature range of bone heated under oxidising conditions. Using these definitions allows for the most accurate representation of the full range of physical and chemical changes associated with both heating conditions.

Understanding the taphonomy and preservation of heated bone is an important prerequisite for interpretations of hominin fire-related behaviour. While microbial decay is the dominant cause of loss of organic materials from the archaeological record, exposure to heat makes bones very resilient against this type of degradation^25,26^. Therefore, the most important factor in heated bone taphonomy is chemical weathering (i.e., diagenesis). However, the diagenesis of heated bone is not well understood. When heated bones enter the depositional environment, they are exposed to different chemical processes that can result in contamination, alteration, and degradation^27^. The specific effect is dependent on the chemical signature of the depositional environment as well as on the physical and chemical properties of the buried material. Diagenesis will therefore affect materials heated to low or high temperatures, and heated with or without oxygen, in different ways. This implies that diagenesis can cause a preservation bias and affect the properties that are used to reconstruct fire temperature and oxygen availability. While there are studies exploring fire as a diagenetic agent affecting bone^28^, and several studies focussing on the diagenesis of charcoal and ash (e.g.,^29,30^), and unheated bone (e.g.,^25,26^), comprehensive research on the effect of diagenesis on heated bone is lacking.

This paper presents the results of laboratory-based experiments into the effect of different pH values as proxies for depositional conditions on the physical and chemical properties of heated bone. The study aims to provide data on (1) the effect of pH-exposure and the processes underlying it, (2) the preservation potential of heated bone in different contexts, and (3) the reliability of the analytical techniques used to reconstruct past heating conditions and fire use. By focussing on pH and taking a controlled experimental approach the study aims to provide an analytical toolkit and reference data that are applicable to archaeological heated bone from any context and time period.

## Methods

The study is based on experiments performed on a total of 107 samples (see Table 1). Two sets of uniform bone samples (cortical bone, bovid) were heated to a range of different temperatures (20–900 °C), one under reducing conditions (i.e., charred) and one under oxidising conditions (i.e., combusted). The resulting samples were incubated for a period of 4 weeks (28 days) in pH solutions representing acidic (pH 3: 0.1 M acetic acid + few drops of HCl), neutral (pH 7: deionised water), and alkaline (pH 12: 0.1 M ammonium hydroxide + 10 nM KOH) conditions (1:10 solid-solution ratio). Note that these specific pH values were not chosen to represent ‘real life’ conditions, but are purposefully more ‘extreme’ to help accelerate the reaction. The experiment was performed in triplicate, with single control samples for pH 7. Samples were incubated in water baths and kept at a constant temperature of 60 °C and 20% vibration (set on the internal scale of the device). Solutions were refreshed twice a week to recreate the setting of an open system with permeable soils and groundwater flow, which enables the introduction of new reagents for the samples to interact with. In order to simulate greater time depth, the reaction was accelerated by increasing the incubation temperature to 60 °C and by using more extreme pH values than are typically found in natural settings. Following the Arrhenius equation, for every increase of 10 °C the reaction speed is doubled. Assuming a normal mean soil temperature of 10 °C, increasing the incubation temperature to 60 °C adds a factor 32 (× 32) to the reaction speed. Since pH is measured on a logarithmic scale, every full value the pH is made more extreme a factor 10 (× 10) is added to the reaction speed (related to the amount of OH^−^ and H^+^ ions). Assuming pH 6 and pH 9 are the ‘normal’ values in nature, using pH 3 and pH 12 solutions results in a factor 1000 (× 1000) to the reaction speed. It should be noted that there are other factors influencing the reaction speed whose precise impact is unknown or cannot be quantified. These include the actual reaction speed of the tested chemical interactions, the effect of incubating whole samples (as opposed to powders), the effect of vibration, and the effect of refreshing the solutions. The quantifiable variables in the experimental setup give a rough estimation in the order of magnitude of 2000–3000 simulated years of pH exposure. After incubation the samples were dried and weighed, before further processing for chemical analysis. For more details on the experimental setup the reader is referred to the Supplementary Information Sections SI 1.1—1.3.

**Table 1:** Overview of the samples used for the preservation experiments.

pH	20	200	300	400	500	600	700	800	900
**Charred**									
3	3	3	3	3	3	3	3	3	3
7	1	–	1	–	–	–	1	–	–
12	3	3	3	3	3	3	3	3	3
**Combusted**									
3	–	3	3	3	3	3	3	3	3
7	–	–	1	–	–	–	1	–	–
12	–	3	3	3	3	3	3	3	3
						Total unheated			7
						Total charred			50
						Total combusted			50
						Full total			107

pH 3 and pH 12 experiments were carried out in triplicate, single samples of selected temperatures were incubated in pH 7 solutions as control samples.

The samples were described for evidence of discolouration, fragmentation, and mass loss. The physical and chemical properties of the samples were analysed using five different analytical techniques: thermogravimetric analysis (TGA), X-ray fluorescence (XRF), Fourier transform infrared spectroscopy (FTIR), pyrolysis gas-chromatography mass spectrometry (Py-GCMS), and X-ray diffraction (XRD). This specific combination of techniques was chosen to target both the organic and inorganic (i.e., bone mineral) content of the bone at different levels (e.g., elemental vs molecular vs structural). The resulting data was analysed and compared with reference data on unexposed charred and combusted bone produced in previous research following the same experiment and analysis protocols (data from Reidsma et al.^23^; van Hoesel et al.^24^). For a more detailed overview of the analytical techniques, their specific settings, and analysis steps the reader is referred to the Supplementary Information Sections SI 1.4–1.7.

## Results

**Figure 1:**
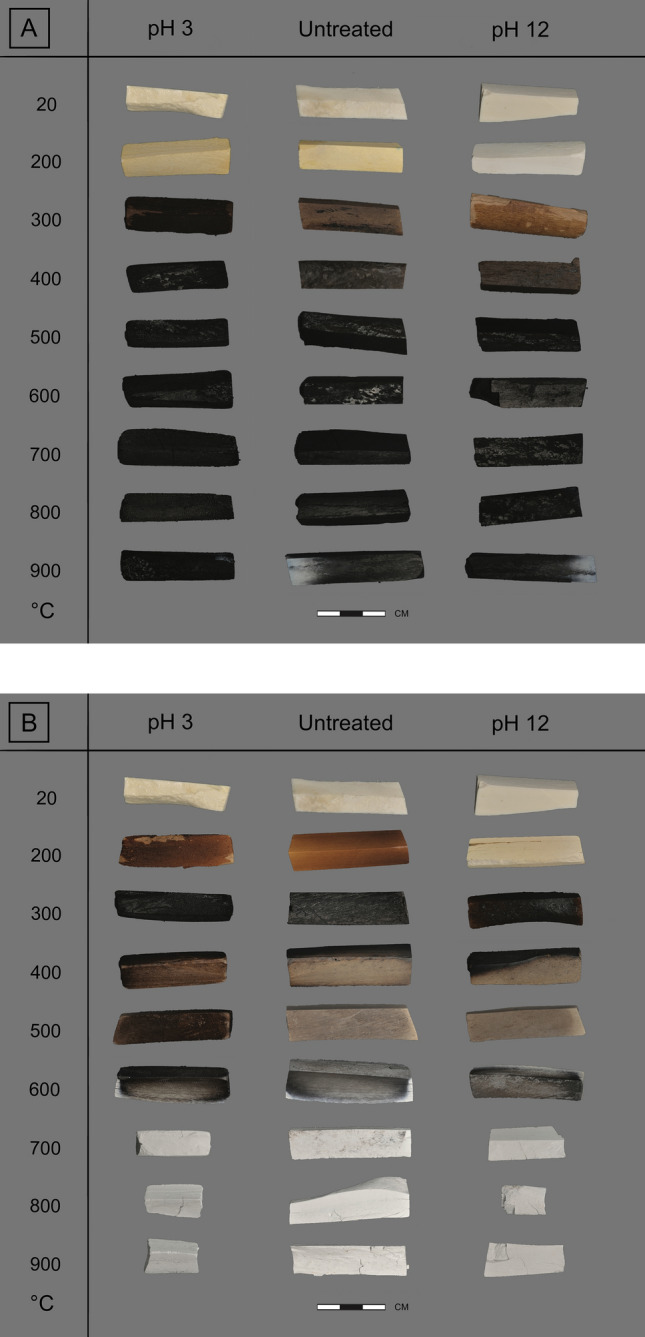
Overview of the variation in colour for charred (**A**) and combusted (**B**) bone exposed to pH 3 and pH 12 conditions. For the control samples exposed to pH 7 conditions (**C**), see the Supplementary Information Fig. SI.2c.

An overview of the results is presented in Tables 2, 3 and 4, and Fig. 1a,b.

**Table 2:** Overview table showing the numeric results for the samples exposed to pH 3 conditions, per analytical technique.

HC	Temp	Colour	Mass loss	TGA			XRF			FTIR		XRD
				Water	Organic	Ash	CaOcorr	P2O5corr	Ca/P	SF	C/P	CI
	°C	Munsell	wt%	wt%	wt%	wt%	wt%	wt%	–	–	–	–
Unheated	20	5Y8/1	15.43	6.91	25.46	65.82	37.00	28.04	1.32	4.32	0.27	0.01
Charred	200	2.5Y8/4	16.80	4.00	14.25	79.71	43.55	35.57	1.22	4.35	0.19	–
Charred	300	5YR3/5	14.87	3.59	10.37	84.02	46.53	36.93	1.26	4.61	0.15	–
Charred	400	5YR2/2	9.02	3.37	8.92	85.75	46.53	38.63	1.20	4.90	0.12	–
Charred	500	5YR2/2	7.92	4.27	8.55	85.66	45.74	39.23	1.16	5.18	0.12	0.07
Charred	600	5YR2/1	6.51	4.21	7.89	86.40	45.35	40.49	1.12	5.32	0.11	0.15
Charred	700	7.5YR2/0	7.21	4.00	6.86	87.75	47.71	39.42	1.21	5.57	0.10	0.22
Charred	800	7.5YR2/0	5.39	2.89	5.57	90.02	48.84	40.55	1.20	6.09	0.07	0.38
Charred	900	7.5YR2/0	5.39	2.14	3.54	93.42	50.89	41.89	1.21	7.15	0.03	0.72
Combusted	200	10YR7/4	19.84	4.81	12.33	81.18	42.20	38.36	1.10	4.61	0.16	–
Combusted	300	5YR3/4	8.59	5.04	11.95	81.24	41.58	39.09	1.06	4.50	0.18	–
Combusted	400	5YR4/1	6.21	3.99	5.08	89.27	46.92	41.69	1.13	4.90	0.12	–
Combusted	500	5YR5/2	7.05	3.49	3.80	91.15	49.02	41.52	1.18	5.12	0.10	0.21
Combusted	600	7.5YR4/0	4.62	2.43	1.97	94.40	51.62	42.15	1.22	6.02	0.07	0.63
Combusted	700	5YR8/1	8.15	0.62	0.29	98.76	54.23	43.89	1.24	6.67	0.04	1.18
Combusted	800	5YR8/1	7.33	0.58	0.22	98.89	53.61	44.61	1.20	6.44	0.04	1.17
Combusted	900	5YR8/1	7.09	0.47	0.22	98.95	53.34	44.89	1.19	5.83	0.04	1.12

For the XRF results the TGA-corrected values are displayed. A dash (–) indicates that particular samples were not analysed for that technique.

**Table 3:** Overview table showing the numeric results for the samples exposed to pH 12 conditions, per analytical technique.

HC	Temp	Colour	Mass loss	TGA			XRF			FTIR		XRD
				Water	Organic	Ash	CaOcorr	P2O5corr	Ca/P	SF	C/P	CI
	°C	Munsell	wt%	wt%	wt%	wt%	wt%	wt%	–	–	–	–
Unheated	20	5Y8/1	5.74	6.75	22.28	68.49	38.50	28.87	1.33	3.99	0.28	0.00
Charred	200	5Y8/1	20.86	4.14	3.33	89.62	50.20	38.42	1.31	4.28	0.16	–
Charred	300	10YR6/4	15.07	4.26	3.29	89.62	50.24	38.40	1.31	4.34	0.15	–
Charred	400	5YR3/2	2.61	4.66	6.30	86.15	47.06	38.03	1.24	4.15	0.18	–
Charred	500	5YR2/1	− 2.73	4.90	6.76	85.44	46.61	37.68	1.24	4.31	0.17	0.06
Charred	600	5YR2/1	− 4.91	4.97	6.81	85.36	46.67	37.55	1.24	4.27	0.18	0.06
Charred	700	7.5YR2/0	− 2.03	5.19	6.14	85.67	47.75	36.92	1.29	4.64	0.17	0.06
Charred	800	7.5YR2/0	− 3.09	3.79	4.35	89.24	49.62	38.52	1.29	5.01	0.13	0.17
Charred	900	7.5YR2/0	− 1.1	2.27	2.78	93.17	51.31	41.14	1.25	5.39	0.07	0.57
Combusted	200	5Y8/1	18.02	4.84	2.68	89.55	46.98	41.54	1.13	4.12	0.18	–
Combusted	300	5YR4/3	7.08	5.42	8.20	83.51	43.72	38.75	1.13	4.13	0.21	–
Combusted	400	5YR5/2	− 2.25	4.57	3.82	88.53	48.03	39.38	1.22	4.10	0.19	–
Combusted	500	5YR5/2	1.61	4.39	3.20	89.48	49.11	39.27	1.25	4.22	0.17	0.04
Combusted	600	7.5YR4/0	− 0.95	2.51	1.67	93.63	51.41	41.40	1.24	5.19	0.10	0.49
Combusted	700	5YR8/1	1.03	0.87	0.66	97.81	53.96	43.14	1.25	6.53	0.06	1.23
Combusted	800	5YR8/1	1.63	0.71	0.57	98.24	53.14	44.47	1.19	5.99	0.06	1.21
Combusted	900	5YR8/1	0.63	0.61	0.55	98.20	52.92	44.59	1.19	5.52	0.05	1.15

For the XRF results the TGA-corrected values are displayed. A dash (–) indicates that particular samples were not analysed for that technique.

**Table 4:** Overview table showing the numeric results for the samples exposed to pH 7 conditions, per analytical technique.

HC	Temp	Colour	Mass loss	TGA			XRF			FTIR		XRD
				Water	Organic	Ash	CaOcorr	P2O5corr	Ca/P	SF	C/P	CI
	°C	Munsell	wt%	wt%	wt%	wt%	wt%	wt%	–	–	–	–
Unheated	20	5Y8/1	5.64	7.32	23.78	66.80	35.72	30.57	1.17	4.01	0.26	0.00
Charred	300	7.5YR6/4	7.49	4.85	10.06	82.81	44.27	38.02	1.16	4.12	0.19	–
Charred	700	7.5YR2/0	− 5.29	5.04	6.61	86.04	45.80	39.68	1.15	4.78	0.15	0.07
Combusted	300	5YR4/2	2.67	5.38	13.86	78.45	41.98	35.96	1.17	4.21	0.20	–
Combusted	700	5YR8/1	0.95	0.66	0.62	98.21	52.85	44.71	1.18	6.64	0.05	1.27

For the XRF results the TGA-corrected values are displayed. A dash (–) indicates that particular samples were not analysed for that technique.

For a detailed description of the data per analytical technique and all supporting figures and tables the reader is referred to the Supplementary Information Section SI 2.

## The effect of different pH conditions

### Acidic conditions: pH 3

**Figure 2:**
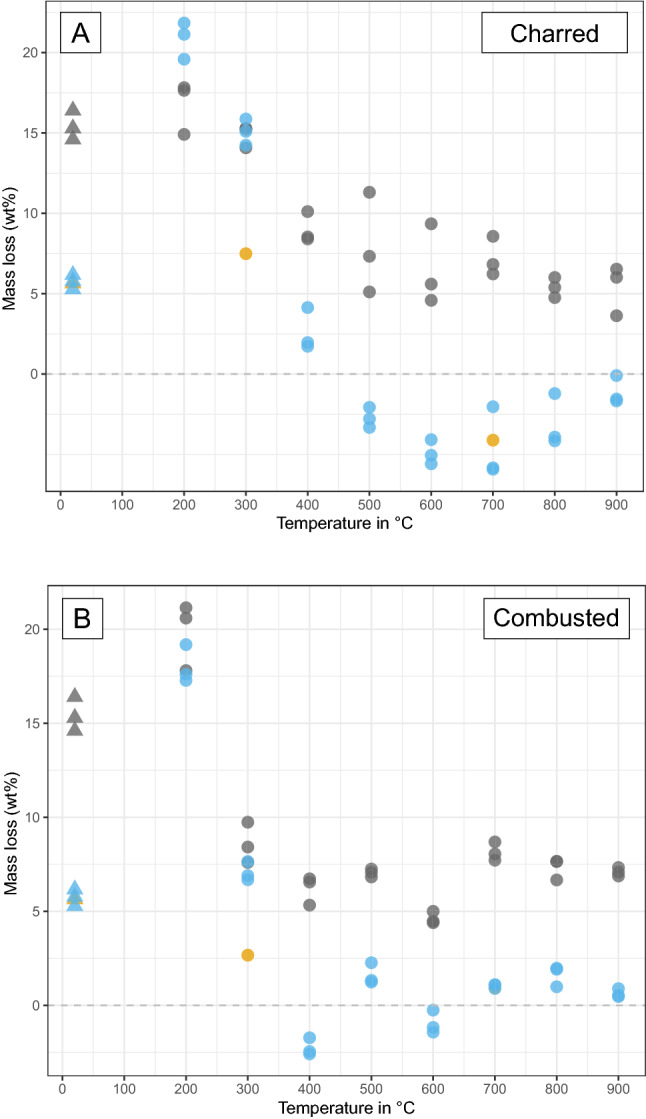
Graphs showing the mass loss (wt%) as a result of pH exposure for charred bone (**A**) and combusted bone (**B**). Triangles = unheated bone, circles = heated bone, grey = pH 3 samples, yellow = pH 7 samples, blue = pH 12 samples, dashed horizontal line = zero mass loss.

**Figure 3:**
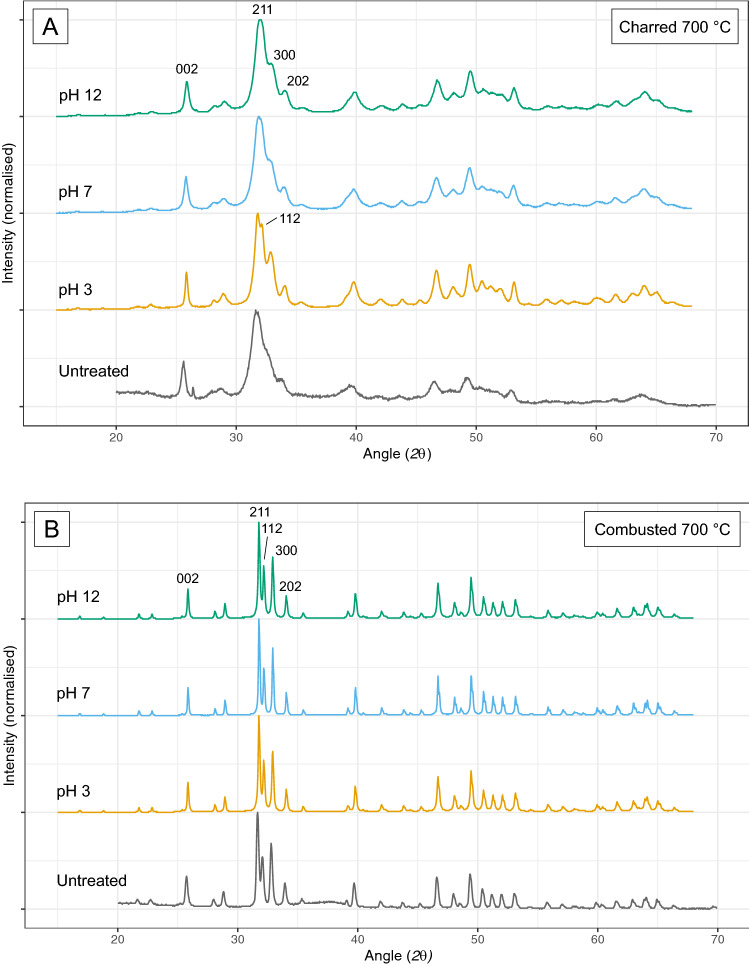
XRD diffractograms for samples charred (**A**) and combusted (**B**) to 700 °C, compared to their untreated equivalent. All notable peaks are labelled. Grey = untreated, yellow = pH 3, blue = pH 7, green = pH 12. For all other diffractograms see Supplementary Information Fig. SI.10a–k.

**Figure 4:**
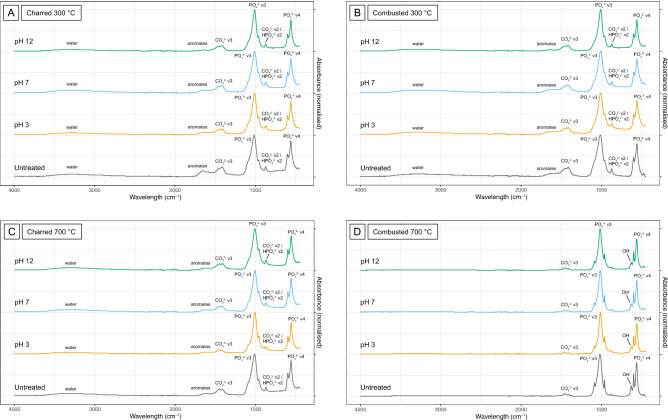
FTIR spectra for samples charred (**A** + **C**) and combusted (**B** + **D**) to 300 °C and 700 °C, compared to their untreated equivalent. All notable peaks are labelled. Grey = untreated, yellow = pH 3, blue = pH 7, green = pH 12. For all other spectra see Supplementary Information Fig. SI.8a–q.

When heated bones are exposed to acidic conditions, the colour of the bones becomes darker compared to unexposed heated bone of the same temperature (Fig. 1a), especially at low to medium temperatures (200–600 °C). Mass loss occurs for the full temperature range, but the effect decreases with increasing temperature (Fig. 2). Acidic conditions cause fragmentation for 53% of the samples, with the most severe effect for bone heated to high temperatures (700–900 °C). Chemical analysis of the samples shows changes to the bone mineral as well as loss of organic material. Exposure to acidic conditions results in the dissolution and loss of carbonates (CO_3_), which is visible in the FTIR data as a reduction of CO_3_ peaks and from decreased C/P ratio values. The XRD data show a sharpening of all peaks in the diffractograms, related to loss of carbonates and some phosphates (PO_4_^3−^) (Fig. 3). In addition, the FTIR data indicates some changes to phosphates: there is increased splitting of the PO_4_^3−^ symmetric stretching and in charred bone the shoulder on the PO_4_^3−^ v3 peak appears at a much lower temperature than in unexposed heated bone (at 400 °C instead of at 900 °C) (Fig. 4 and Figure SI.8). The increase in FTIR-SF and XRD-CI values for all heated bone samples indicates a clear increase in crystallinity of the bone. TGA and Py-GCMS data show that there is also some dissolution and loss of organic materials. This occurs in bone heated to low and medium temperatures (200–500 °C) and only affects specific molecular compound types (i.e., monocyclic aromatic hydrocarbons (MAH), methylene chain compounds (MCC), polycyclic aromatic hydrocarbons (PAH), and tert-butylphenols (TERT)).

Exposure to acidic conditions has a similar effect on charred and combusted bone, but the impact is a little more severe for charred bone. The data show a general trend of more mass loss for charred bone, especially at low to medium temperatures (200–500 °C). This trend is matched in the TGA data, which shows more loss of organic compounds for charred bone. The effect of exposure to pH 3 solutions is not intense enough to override the difference caused by the difference in oxygen availability between charred and combusted bone (e.g., see FTIR spectra).

The same processes that affect heated bone under acidic conditions also affect unheated bones. However, unheated bone is affected less severely than heated bone, especially when compared to low temperature heated bone (200–300 °C), which is chemically most similar to unheated bone. Compared to the other pH values, unheated bone is most affected by exposure to pH 3 (Figs. 1 and 2).

### Alkaline conditions: pH 12

**Figure 5:**
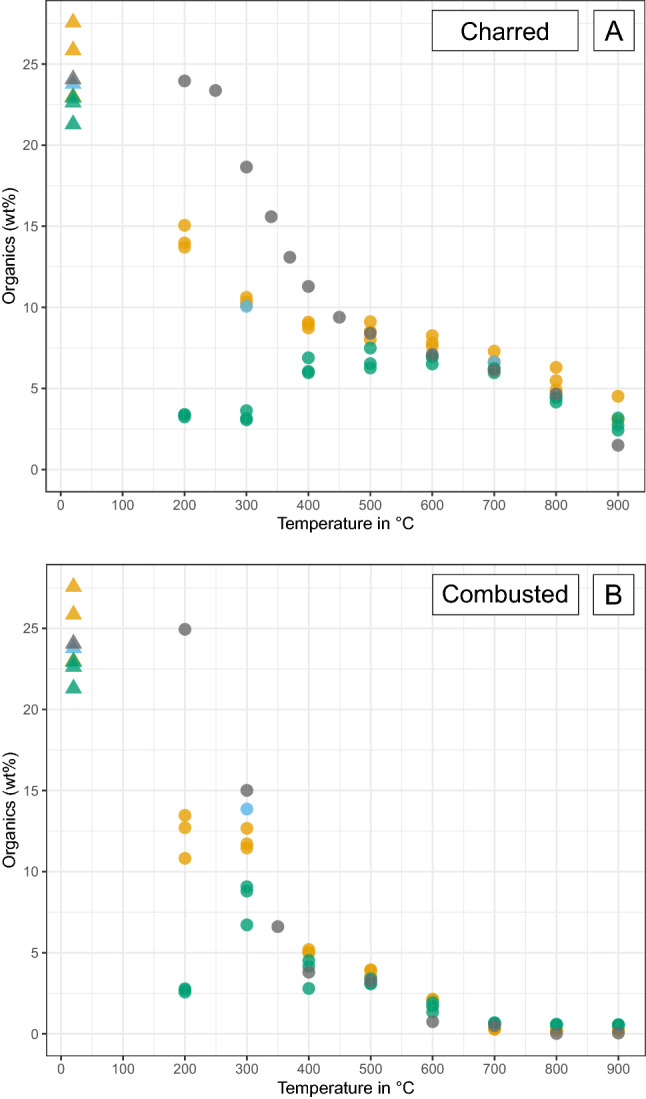
Graphs showing the variation in organic content (wt%) in charred (**A**) and combusted (**B**) bone, as measured by TGA, as a result of pH exposure. Triangles = unheated bone, circles = heated bone, grey = untreated samples, yellow = pH 3 samples, blue = pH 7 samples, green = pH 12 samples.

Exposure to alkaline conditions leads to a much lighter bone colour compared to unexposed heated bone of the same temperature (Fig. 1), especially at temperatures of 20–700 °C. Mass loss is most severe for bone heated to 200 °C and decreases with increasing temperature (Fig. 2). Above 400 °C mass loss often dips below zero, indicating a mass increase. Exposure to alkaline conditions causes fragmentation for 53% of the samples with the most severe effect for bone heated to high temperatures (700–900 °C). Chemical analysis of the samples indicates the loss of organic compounds. TGA results show loss of organic content that is more severe than for exposure to pH 3, but still decreases with increasing temperature (Fig. 5). The loss of organic content is reflected in the FTIR data as a reduction of peaks related to amides and aromatic compounds (Fig. 4). Py-GCMS data indicates that it specifically concerns the dissolution and loss of nitrogen-containing compounds (NCOMP), phenols (PHEN), and triterpenoids (TRITERP). Furthermore, while sensitive to dissolution under pH 3 conditions in low temperature heated bone (200–300 °C), methylene chain compounds (MCC) and polycyclic aromatic hydrocarbons (PAH) become sensitive to exposure to pH 12 conditions in bone heated to higher temperatures (400–500 °C). The nitrogen-containing compounds make up the majority of the organic compounds present in (heated) bone, and many of those compounds are completely lost as a result of exposure to alkaline conditions. This effect is particularly severe for bone heated to 200 °C, and decreases with increasing temperature alongside the heat related decrease in NCOMPs. Phenols are also completely lost in bone heated to 200 °C. In addition, the FTIR and XRD data show changes to the bone mineral. The reduction of the CO_3_ peaks and C/P ratio may indicate loss of carbonates, however, these peaks overlap with organic compounds and reduction as a result of the loss of organic content cannot be ruled out. The effect is always less severe than for heated bone exposed to acidic conditions. Changes to the phosphate are shown by increased splitting of the PO_4_^3−^ v1 symmetric stretching as well as by an increase in FTIR-SF and XRD-CI values for all temperatures. However, overall, these changes are less severe than for heated bone exposed to acidic conditions.

Exposure to alkaline conditions has a similar effect on charred and combusted bone, but the impact is more severe for charred bone. The data show a general trend of more mass loss for charred bone, especially at low to medium temperatures (200–500 °C). TGA data also shows more loss of organic compounds for charred bone than for combusted bone. The effect of exposure to pH 12 solutions is so severe for bone heated at low to medium temperatures (200–500 °C) that it overrides the differences created by the difference in oxygen availability, rendering the chemical signature of both charred and combusted bone at these temperatures very similar (e.g., see FTIR spectra).

The same processes that affect heated bone under alkaline conditions also affect unheated bones. However, unheated bone is affected less severely than heated bone, especially when compared to low temperature heated bone (200–300 °C), which is chemically most similar to unheated bone. Unheated bone is much less affected by pH 12 than by exposure to pH 3 (see Figs. 1 and 2).

### Neutral conditions: pH 7

To control for the effect of neutral conditions samples of selected temperatures were used (n = 5, see Table 1). Exposure to neutral conditions has a limited effect on heated bone colour, with only the charred bone samples displaying a change to a lighter colour. There is mass loss for all samples, which decreases with increasing temperature (Fig. 2). Chemical analysis of the samples indicates that these changes relate to the loss of organic compounds and some carbonates. There is also a mild increase in crystallinity, as evidenced by increased FTIR-SF and XRD-CI values. All changes occur to a lesser extent than for samples exposed to pH 3 or pH 12 solutions.

Combusted bone appears to be less affected by exposure to neutral conditions than charred bone. There is no change in colour for combusted bone, and mass loss is much lower than for charred bone (Fig. 2). However, the effect on the crystallinity (FTIR-SF and XRD-CI) of the bone mineral is slightly more severe for combusted bone than for charred bone.

The effect of neutral conditions is even more limited for unheated bone. Bone colour becomes lighter and there is mass loss similar to that of unheated bone exposed to pH 12 conditions. However, while FTIR data indicates loss of organic compounds and carbonates, TGA gives organic content values similar to unexposed bone.

## Processes underlying pH exposure

Based on the pH-specific data some inferences can be made about the processes underlying pH exposure. The two main processes affecting heated bones as a result of pH-exposure are dissolution of the remaining organic and inorganic compounds and re-organisation of the apatite crystal structure. Dissolution, or breakdown, occurs when molecules become soluble under specific conditions. This is the case for organic molecules (e.g., collagen and its thermal products) in pH 12 conditions^25,31^, and for carbonates and phosphates in pH 3 conditions^31,32^. The affected compounds dissolve and become suspended in the solution, causing them to leach out of the bone. Under acidic conditions the process of hydrolysis can break down the bonds in certain organic molecules as well (e.g., collagen-derived products) and render the resulting fragments soluble under those conditions^33,34^. This process is likely what caused the loss of some organic compounds from the heated bones exposed to acidic conditions. Additionally, the loss of some hydrocarbons (e.g., alkenes) under pH 3 conditions is likely the result of their solubility in organic solvents, such as acetic acid. The shift from loss of PAHs in pH 3 conditions at low temperatures (200–300 °C) to loss of PAHs in pH 12 conditions at higher temperatures (400–500 °C) might be related to formation of PAHs with higher molecular weight at these temperatures, which is known to affect their solubility^35^. However, more research is needed to confirm this. Dissolution thus results in loss of organic compounds and/or carbonates and phosphates, and in turn facilitates the re-organisation of bone mineral into a more crystalline material. The loss of material frees up space for recrystallisation products. This process is visible in the data as an increase in SF and CI values as a result of exposure to both pH 3 and pH 12 conditions.

A very tangible effect of dissolution as a result of pH exposure is discolouration of heated bone. The data show that dissolution of organic compounds (dark coloured) under pH 12 conditions results in lighter coloured bones, while dissolution of inorganic compounds (light coloured) under pH 3 conditions results in darker coloured bones. This further confirms that heated bone colour is primarily governed by the presence, state, and amount of organic content. Fragmentation of heated bone, while affected by pH exposure, appears to be primarily related to alteration and loss of material through heating. In unheated bone, it is the presence of collagen that gives the bone its flexibility^36^. Therefore, the continued presence of unaltered collagen is likely what prevents the occurrence of pH-mediated fragmentation in unheated bone. However, this changes when the organic compounds in bone become thermally altered, decrease, and ultimately disappear at high temperatures. The results show that at 200–600 °C, dissolution and loss of organic or inorganic material both cause destabilisation of the bone that facilitates fragmentation. At 700–900 °C, when apatite crystallinity is high and organic content low or non-existent, fragmentation is not caused by dissolution. Instead, the high percentages of fragmentation likely relate to the already fragile state of high temperature heated bone, coupled with available moisture seeping into existing micro-fissures and cracks, causing fragmentation irrespective of pH^37^.

Another pH-related effect is the mass increase (or negative mass loss) for certain heated bone samples exposed to pH 12 conditions, being particularly prevalent for charred bone. This effect relates to increased water retention, particularly in charred organic matter, as indicated by the TGA data. The data show consistently higher wt% values for water content for samples exposed to pH 12 solutions. The removal of soluble organic compounds likely increased the microporosity of the bone, resulting in increased water retention even after storage in a desiccator and prolonged drying at 60 °C. Other explanations, such as adsorption of carbonates or the binding of CO_2_ from the surrounding environment, were considered, but do not fit the data. The setup of the incubation experiments was specifically designed to prevent CO_2_ absorption through the use of closed vials, and no carbonates were introduced in the solutions.

## Discussion

### Preservation

The results clearly describe the effect of pH exposure on the preservation of heated bone in the archaeological record. As heated bone, charred or combusted, is more resilient to microbial attack, burial conditions and especially soil pH are the most important factor for its preservation potential. Fragmentation occurred for over 50% of the samples exposed to pH 3 and pH 12 solutions, and to 40% of the samples exposed to pH 7. Strikingly, no fragmentation occurred for the unheated bone samples, showing that heated bone is more friable than unheated bone regardless of pH or heating temperature. Other taphonomic factors may increase the fragmentation rate to such an extent that the heated bone becomes difficult to recognise or is lost all together. This might especially be the case for high temperature heated bone, as the fragmentation rate increases with increasing heating temperature. This could then cause a preservation bias against bone heated to high temperatures (700–900 °C). Material is also lost through dissolution of organic compounds, carbonates and phosphates. Loss of these compounds destabilises the bone and promotes other forms of degradation, such as microbial decay and fragmentation. Dissolution is particularly severe for low temperature heated bone (200–400 °C), and can potentially cause a preservation bias against these temperatures. Within the current experimental setup, several of the nitrogen-containing compounds (i.e., related to collagen, e.g., diketodipyrrole) are already completely lost as a result of exposure to alkaline conditions. For this study samples of dense bovid cortical bone were used. This type of bone is very robust, and although roughly 2000–3000 years were simulated, the effect of pH exposure alone already has a significant effect on the preservation potential of heated bone. These results can therefore be used as a baseline for minimal bone decay, as the effects will likely be more severe for heated bone when different variables are considered. Bone with a lower density, such as trabecular bone or bones from small mammals, will have a much lower preservation potential. In addition, the effect of pH exposure might be more severe for more prolonged exposure, such as for Palaeolithic time scales. Finally, the rate of degradation and loss may increase in combination with other post-depositional processes, such as microbial attack, frost action, and general formation processes. The influence of pH exposure on the preservation potential of heated bone is also dependent on the moisture content, amount of buffering material (e.g., alkaline: ash, shells, carbonate rich mud; acidic: podzols, pyrite oxidation), and openness of the soil geochemical system. Mitigating factors that will promote the preservation of heated bone are dry conditions (i.e., absence of water)^38,39^ and low ion activity in flow and diffusion restricted environments such as in clay sediments^40^.

### Reconstructing heating conditions

Exposure to different depositional environments affects both the preservation potential of heated bone and the parameters that are used to reconstruct heating conditions (i.e., temperature and oxygen availability). Bone colour is frequently used as a proxy for heating temperature (e.g.,^41–43^). However, this study demonstrates that bone colour is not only dependent on heating conditions, but also affected by pH. At low to medium temperatures (200–500 °C) bone colour is altered by exposure to acidic and alkaline conditions. The pH-induced colour changes may therefore cause misinterpretation of heating temperatures, for example a light-coloured bone heated to 200 °C exposed to alkaline conditions can be mistaken for bone combusted at high temperature (700–900 °C).

Heating conditions are also reconstructed using analytical techniques such as TGA, FTIR, and XRD (e.g.,^44–50^). The reliability of the outcomes of these techniques can be severely affected by pH exposure depending on what specific part of the bone they target. TGA can be used to reconstruct heating conditions by tracking the changes in wt% of organic content. This means that the technique is affected by loss of organic material, but also by loss of bone mineral, as this results in a relative increase in organic content. Since most organic material is present in heated bone at low to medium temperatures (i.e., < 600 °C), the pH-related offset is largest for those temperatures and TGA is more reliable for high temperatures. If left uncorrected, TGA values for bone heated to 200 °C and exposed to pH 12 might be misinterpreted for bone heated to much higher temperatures (e.g., combusted to 500–600 °C; charred to 800–900 °C).

FTIR targets the molecular content of bone and can be used to track both changes in organic content and in bone mineral. FTIR can be used to reconstruct heating conditions by comparing spectra (i.e., presence, shape, intensity of peaks) and by calculating indices such as the Splitting Factor (SF). Both options are affected by pH exposure, resulting in increased SF values and spectra that resemble those of different heating conditions. For example, spectra for bone heated to low and medium temperatures (200–500 °C) and exposed to alkaline conditions become identical for charred and combusted bone.

XRD can be used in a similar way as FTIR, but only targets the inorganic components of the bone. Peak shape and intensity are minimally affected by exposure to pH 3, while exposure to both acidic and alkaline conditions results in increased Crystallinity Index (CI) values. This could result in an overestimation of the heating temperature, especially for lower temperatures, where the effect of pH is most severe.

To summarise, the results of this study show that exposure to acidic conditions mainly affects the reliability of methods targeting the bone mineral, while exposure to alkaline conditions mainly affects the reliability of methods targeting the organic content. In both cases the effect decreases with increasing temperature, making the techniques more reliable for high temperature heated bone (700–900 °C) than for bone heated at low temperatures (200–600 °C). However, irrespective of specific pH conditions, the survival of high temperature heated bone is much lower than that of its low temperature equivalent due to high rates of fragmentation.

### Implications

The results of this study have implications for the way fire traces, and specifically heated bones, are studied in the archaeological record. Firstly, the demonstrated effect of exposure to different pH conditions on the preservation potential of heated bone suggests a re-evaluation of the patterns of presence and absence of fire traces and their resulting interpretations is warranted. The data on dissolution and loss of certain compounds (e.g., phenols, PAHs, collagen) also provides clues into their preservation potential in other heated materials or outside of bone (e.g., biomarkers in sediments). The difference between the effect of pH exposure on heated versus unheated bone implies that the presence of unheated bone at an archaeological site is not necessarily an indication that heated bone should be preserved, and vice versa. Furthermore, loss of specific compounds can impact the reliability and success rate of other types of chemical analyses, such as isotope studies, C14 dating, and the study of biomarkers and aDNA.

Secondly, the demonstrated effect of pH exposure on the reliability of the outcome of the different methods to reconstruct heating conditions suggests that a re-evaluation of existing studies addressing fire temperature and oxygen availability is also warranted. This study shows that it is essential to take the effect of diagenesis into account when reconstructing heating conditions. This involves using pH-specific reference data as well as measuring the pH and other geochemical signatures of the sediments (e.g., see^20,51^). The latter can also provide valuable insight into the preservation conditions of unheated materials and help explain their distribution or absence.

While the results of this study provide significant insights into the diagenesis of heated bone and its implications, there are some limitations that briefly need to be addressed. The nature of a controlled experimental setup dictates that only a specific set of variables are tested. Therefore, the effect of variation in exposure time, type of bone, and fluctuating conditions were not part of this study. In addition, practical constraints resulted in a very limited samples size to test the effect of exposure to neutral conditions. Nevertheless, the combination of tested variables provides a solid base to understand the effect of pH-exposure on heated bone. The limitations of the experimental setup do have an impact on the application of the reference data to archaeological material. This is the case in particular because diagenesis is a gradient and the data presented here only provide one reference point on the spectrum between perfectly preserved and fully degraded bone. Future research should therefore include complimentary experiments testing the effect of pH-exposure in conjunction to other variables. However, in the absence of further experimental work, the pH reference data presented here provides a significant step up in the accuracy with which heating conditions can be reconstructed from archaeological heated bone, by explicitly taking the effect of diagenesis into account.

**Figure 6:**
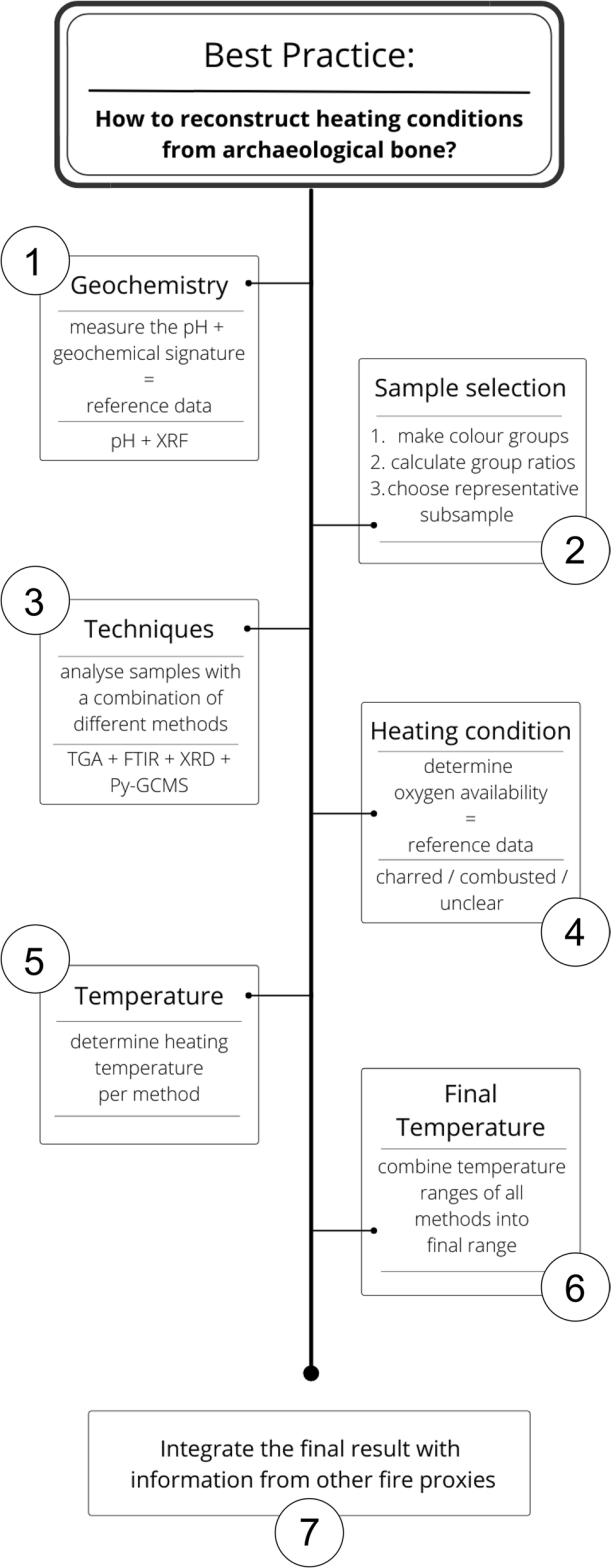
Flow chart displaying the best practice for the study of archaeological heated bones for fire-related research questions, explicitly taking the effect of diagenesis into account.

Presented here is a best practice for the study of archaeological heated bones for fire-related research questions (Fig. 6). The process should start with determining the pH (and geochemical signature) of the site, preferably throughout the sequence and both within and outside of the bone sampling context (e.g., hearth features). The resulting data is used to inform the choice of reference data used for the chemical analysis (i.e., reference data for acidic, neutral, or alkaline conditions). Ideally the bone samples are sieved out of the bulk sediment samples used for the pH and geochemical analysis. Alternatively, bone samples can be retrieved from the 5 mm mesh general sieving residue. Selecting small fragments allows for a more representative sample, accounting for the increased fragmentation of high temperature heated bone. The bone fragments should then be sorted into colour classes (e.g., light vs. dark). While bone colour is not a reliable indicator of heating conditions, it is a good proxy for potential differences in chemical composition. Based on the different colour classes, a representative subsample is selected for further analysis. The samples should be analysed using a combination of techniques that target different aspects of the bone, e.g., organic and inorganic compounds, as well as low and high exposure temperatures. A combination of TGA, FTIR, XRD, and Py-GCMS works well for this purpose. Based on the analysis results the oxygen availability and heating temperature are determined per technique, using the relevant reference data (i.e., specific pH; charred, combusted, or both). Finally, the results of the different methods are combined to give the most likely temperature range, which can then be integrated with information from other heated materials and contribute to the record of and the resulting interpretation about fire use at the site.

## Conclusion

The current study provides insight into the effect of diagenesis on fire proxies within different depositional contexts by investigating the effect of pH exposure on the physical and chemical properties of heated bone. The experiments serve as a baseline for further investigations into the taphonomy of fire remains. The main conclusions from the study are:

- Exposure of heated bone to different pH values causes discolouration, fragmentation, and mass loss. Exposure to acidic conditions results in darker colours, while exposure to alkaline conditions results in lighter colours. For both pH values fragmentation occurred in over 50% of the samples. The most severe fragmentation occurs for bone heated to high temperatures (600–900 °C).
- Heated bone is more friable and susceptible to degradation than unheated bone (but more resilient to microbial decay), regardless of pH and without the influence of other taphonomic factors. The presence of unheated bone at a site is not an indicator for the preservation of heated bone, and vice versa.
- The degree of degradation is dictated by the properties of the bone before entering the archaeological record. Because of the more homogenous and chemically stable nature of high temperature heated bone, there is a decrease in the effect of pH exposure with increasing heating temperature. The most severe chemical changes occur at low to medium temperatures (200–500 °C).
- Due to the difference in physical and chemical properties of charred versus combusted bone, pH exposure has a more severe effect on charred bone than on combusted bone.
- Exposure to acidic conditions mainly affects the bone mineral, causing loss of carbonates and phosphates and an increase in crystallinity. Organic compounds like monocyclic aromatic hydrocarbons, methylene chain compounds, polycyclic aromatic hydrocarbons and tert-butylphenols are also lost.
- Exposure to alkaline conditions mainly affects the organic content of the bone, causing loss of collagen and char, and also an increase in crystallinity. The majority of the organic compounds in heated bone are lost as a result of exposure to alkaline conditions (nitrogen-containing compounds, phenols, and triterpenoids).

This study highlights the need for studies of ancient fire use to explicitly take diagenesis into account, and provides the toolkit and reference data to do so.

## Supplementary Information

Supplementary Information.

## Data Availability

All data generated and used to support the claims made in this paper are presented here and in the Supplementary Information. The complete raw datasets for this study, as well as for previous work on charred and combusted bone will become available on Zenodo after the embargo period has concluded. The datasets will be available under the following DOIs: Heated bone pH reference data: https://doi.org/10.5281/zenodo.6772968. Charred bone reference data: https://doi.org/10.5281/zenodo.6772889. Combusted bone reference data: https://doi.org/10.5281/zenodo.6772924.
