# Supplementary material for: Laboratory-based experimental research into the effect of diagenesis on heated bone: implications and improved tools for the characterisation of ancient fire
Source: Sci Rep. 2022 Nov 2;12:17544. doi: 10.1038/s41598-022-21622-5 (PMC9630262; doi:10.1038/s41598-022-21622-5)
Supplement: Supplementary file 1 — Supplementary Information. [file 41598_2022_21622_MOESM1_ESM.pdf]

SUPPLEMENTARY INFORMATION

Laboratory-based experimental research into the effect of diagenesis on heated bone: implications and improved tools for the characterisation of ancient fire

Femke H. Reidsma[1,2]*

1 – Human Origins Group, Faculty of Archaeology, Leiden University, Leiden, The Netherlands
2 – GeoLab, Faculty of Geosciences, Utrecht University, Utrecht, The Netherlands

*Corresponding author: f.h.reidsma@arch.leidenuniv.nl

SI 1: Materials and Methods

SI 1.1 Sample preparation

Samples were produced from several mature female bovine (*Bos taurus*) femurs obtained from a local butcher. The epiphyses were cut off with a handsaw and any remaining flesh, fat, and marrow was mechanically removed, after which the bones were further cleaned with water (<40 °C) and air-dried. Cleaned cortical bone was cut into longitudinal samples measuring approximately 45 x 10 x 10 mm, using a water-cooled precision saw equipped with a diamond wafer blade (Buehler IsoMet® 1000 Precision Saw). Bovine bone was chosen as an analogue for the large herbivores that dominate most Pleistocene assemblages, as well as to match earlier experimental work by the author.

SI 1.2 Heating

Samples were individually heated to the required temperatures (200, 300, 400, 500, 600, 700, 800, and 900 °C), and placed in a preheated furnace for 60 minutes. Heated bone samples were produced under two different atmospheric conditions: with and without oxygen (i.e., combusted vs. charred). Charring was done under controlled conditions using a Carbolite MTF 12/38/250 tube furnace. Individual samples were placed in an open Pyrex© vessel and inserted into a 30 cm long Pyrex© tube at 18 cm from the inlet, which was then inserted into the furnace. Heating was done at atmospheric pressure and under constant nitrogen flow (150 ml/min) (cf. [SI.1]). Volatiles were vented and trapped in liquid DCM. After heating the samples were left to cool under continued nitrogen flow until they could safely be handled and further cool without nitrogen. Combustion was done under controlled conditions using a Carbolite GVA 12/300 tube furnace. A stainless-steel work-tube was used. Individual samples were put in a ceramic vessel and placed on a metal mesh platform attached to a handle with metal rods. Right below the handle some glass wool insulation material was placed between two additional pieces of metal mesh (Figure SI.1). This set up was designed to allow the samples to be placed in the middle of the vertical tube furnace, and to allow enough airflow through the furnace (to ensure combustion) while keeping the temperature inside the furnace stable. Before samples were inserted, the furnace was allowed to heat up to the required temperature and stabilise for an hour (or longer, if needed). Because of the semi-open set up, the temperature in the furnace was checked with a thermocouple at the level of the samples (TME MM2020 dual input thermocouple thermometer + RÖSSEL MESSTECHNIK thermocouple type K K1.2), and adjusted where necessary. Samples were left to cool outside the furnace.

SI 1.3 Incubation

Samples (n = 107, Table SI.1) were incubated in pH solutions for a period of 4 weeks (28 days). Individual samples were placed in 50 ml tubes (Sarstedt polypropylene self-standing centrifuge tubes), to which 45 ml of pH solution was added to represent acidic, neutral, or alkaline conditions (solid-solution ratio 1:10). Acidic conditions (pH 3) were created using a 0.1 M acetic acid solution,

with a few drops of HCl. For the Alkaline conditions (pH 12) a solution of 0.1 M ammonium hydroxide and 10 nM KOH was used. Neutral conditions were created using only deionised water. The samples were incubated in closed tubes to avoid additional chemical reactions with CO_2 and O_2 from the air. Samples were incubated in a water bath (a GFL 1083, and two New Brunswick Scientific Gyrotory water bath shakers model G76) and kept at constant temperature of 60 °C and 20% vibration (set on the internal scale of the devices). Solutions were refreshed twice a week, and the pH was checked using an Accumet AB150 benchtop pH meter equipped with a double junction pH/ATC electrode. The solutions were mixed using primarily a weak acid and a weak base to ensure the availability of enough ions for a reaction in between refreshing. The solid-solution ratio (1:10) was also chosen to ensure the availability of enough reagent in between refreshing. This was confirmed by measuring the pH before and after incubation. While the pH of the solutions changed as a result of interaction with the samples, the solutions maintained a pH within the range associated with acidic, neutral, and alkaline conditions. This set up was chosen to exert control over the variables involved, to accelerate the reaction, and to mimic soil conditions. The vibration and refreshing of solutions were done to recreate the context of an open system with permeable soils and potential groundwater flow, bringing in new reagents for the material to interact with. By increasing the incubation temperature to 60 °C and using more extreme pH values than are typically found in natural settings, reaction times were shortened, simulating a prolonged time depth compared to a natural situation. A rough estimation of the simulated time depth can be made based on the following steps. Following the Arrhenius equation, for every increase of 10 °C the reaction speed is doubled. Assuming a normal mean soil temperature of 10 °C, increasing the incubation temperature to 60 °C adds a factor 32 (x32) to the reaction speed. Since pH is measured on a logarithmic scale, every full value the pH is made more extreme a factor 10 (x10) is added to the reaction speed (related to the amount of OH^- and H^+ ions). Assuming pH 6 and pH 9 are the 'normal' values in nature, using pH 3 and pH 12 solutions results in a factor 1000 (x1000) to the reaction speed. It should be noted that there are other factors influencing the reaction speed whose precise impact is unknown or cannot be quantified. These include the actual reaction speed of the tested chemical interactions, the effect of incubating whole samples (as opposed to powders), the effect of vibration, and the effect of refreshing the solutions. Therefore, an exposure time of 4 weeks gives a rough estimation in the order of magnitude of 2000-3000 simulated years. It should be noted that only the increase of temperature affects the reaction speed of the samples exposed to pH 7 solutions. After incubation all samples were dried in a VWR Dry-Line oven at 60 °C and weighed daily until no further mass loss occurred, after which they were left to acclimatise for 24 hours.

SI 1.4 Physical analysis

SI 1.4.1 Colour

Bone colour was determined on powdered material using the Munsell Soil Color chart [SI.2], as well as through photographs taken before and after incubation.

SI 1.4.2 Fragmentation

The degree of micro and macro fragmentation resulting from incubation was assessed visually by noting presence of floating particles (micro) at each refreshing, and by counting the number of fragments (macro) after the full experiment.

SI 1.4.3 Mass loss

All samples were weighed before and after incubation to determine the mass loss. Weighing was done after cooling/drying and acclimatisation, and before powdering. Weighing after incubation included all macro fragments of the original sample.

SI 1.4.4 Thermogravimetric analysis (TGA)
TGA was performed on 1 g of powdered sample, using a LECO TGA 701 machine. Samples were heated from 25 to 1000 °C with a heating rate of 5 °C/min. Air was used as the carrier gas to ensure complete combustion of the samples. The water content was determined as the percentage of mass loss at 200 °C. The organic content was determined as the percentage of mass loss between 200 and 600 °C, and the ash content as the remaining mass percentage at 950 °C.

SI 1.5 Elemental analysis

SI 1.5.1 X-ray fluorescence (XRF)
The XRF analyses were carried out using a Thermo Scientific Niton XL3t GOLDD+ energy-dispersive p-XRF analyser, equipped with a silicon drift detector. Powdered samples were compressed manually with an agate pestle into a PE-ring with a height of 10 mm. Analyses were performed using a stand mounted above the sample ring. This set-up negates the use of separation foil between the sample and the XRF, and avoids absorption of secondary X-rays from light elements. Measurements were taken in Cu/Zn-mining mode, with a measuring time of 110 sec, using 4 sequential energy settings: Light range (Mg to Cl) at 8 kV 200 µA, low range (K to Ti) at 20 kV 100 µA, main range (V to Ag including L-lines for Pb) and high range (Cd- Ba) both at 50 kV, 40 µA. Factory calibration was checked and adjusted using a set of 14 powdered ISE standard soil samples (www.wepal.nl). Accuracy was tested using the BAMS005B glass standard. CaO and P_2O_5 Values were further corrected using the TGA data and the following formula, taking into account that the sum of organic and inorganic compounds equals 100%:

$$CaOcorr = \left(\frac{CaO}{total\ inorganic\ XRF}\right) \times total\ inorganic\ TGA$$

SI 1.6 Molecular analysis

SI 1.6.1 Fourier transform infrared spectroscopy (FTIR)
Single measurements were taken on powdered material using an Attenuated Total Reflectance (ATR) Perkin Elmer Spectrum100 FT-IR spectrometer, equipped with a diamond ATR crystal. Absorption spectra were accumulated in 16 scans for the 4000-450 cm^{-1} wavelength range, with a spectral resolution of 4 cm^{-1}. All spectra were background corrected and normalised to the highest peak. The FTIR splitting factor (SF) was calculated following Weiner and Bar-Yosef [SI.3]. The C/P ratio was calculated using the height of the carbonate peak at 1417 cm^{-1} (CO_3^{2-} v3) and the phosphate peak at 1015 cm^{-1} (PO_4^{3-} v3).

SI 1.6.2 Pyrolysis gas-chromatography mass spectrometry (Py-GCMS)
For the Py-GCMS single measurements were taken on powdered material from two out of the three replicated samples (e.g., sample A2 + C2, 266 + 267, etc.). For the samples exposed to pH 7 conditions, where no replications were available, analytical replications were obtained by measuring the samples twice. Due to measurement issues for sample G2 sample E2 was also measured twice. A Pyroprobe 5000 (CDS Analytical) coupled to an Agilent 6890/5975 GC-MS was used for Pyrolysis-GC-MS analysis. Samples were introduced in quartz tubes, embedded with deactivated quartz wool. Pyrolysis was performed at 650 °C, reached at a rate of 10 °C/ms. The pyrolysis interface, the GC inlet and the GC-MS interface were maintained isothermal at 325 °C. The GC oven was heated from 60 °C (1.5 min isothermal) to 325 °C (3 min dwell time) at a rate of 20 °C/min. Helium was used as a carrier gas at a flow rate of 1 ml/min. The GC was equipped with a HP5-MS (non-polar) capillary column and operated in split mode (1:10). The electron impact (EI) ionization energy of the MSD was 70 eV and the quadrupole detector scanned in the m/z range of 50-400.

SI 1.7 Structural analysis

SI 1.7.1 X-ray diffraction (XRD)

Single measurements were taken on fixed, powdered samples, placed in PMMA sample holders with a diameter of 25 mm and a depth of 1 mm. Analyses were carried out using a Bruker-AXS D8 advance powder x-ray diffractometer configured with LYNXEYE detector and DAVINCI design. The machine is equipped with two circle goniometer theta/2theta, and a ceramic 2.2 kW Cu K $\alpha_{1,2}$ tube (12 mm λ = 1.54060 Å), and is operated at 40 kV and 40 mA. Measurements were collected in the 16-68° 2θ range, with a step size of 0.04° 2θ, a step time of 1 sec, and 17 mm illumination. The gonio radius was 280 mm, and a 2.5° soller slit was used. The Crystallinity Index (CI) was calculated following Person et al. [SI.4].

References

SI.1 Braadbaart, F., Poole, I. & van Brussel, A. A. Preservation potential of charcoal in alkaline environments: an experimental approach and implications for the archaeological record. *J. Archaeol. Sci.* **36,** 1672–1679, doi: 10.1016/j.jas.2009.03.006 (2009).

SI.2 *Munsell Soil Color Charts*. (Munsell color company, inc., 1954).

SI.3 Weiner, S. & Bar-Yosef, O. States of preservation of bones from prehistoric sites in the Near East: A survey. *J. Archaeol. Sci.* **17,** 187–196, doi: 10.1016/0305-4403(90)90058-D (1990).

SI.4 Person, A. *et al.* Early diagenetic evolution of bone phosphate: An x-ray diffractometry analysis. *J. Archaeol. Sci.* **22**, 211–221, doi: 10.1006/jasc.1995.0023 (1995).

Supplementary Information 1: tables

Table SI.1: Overview of the samples used for the preservation experiments. pH 3 and pH 12 experiments were carried out in triplicate, single samples of selected temperatures were incubated in pH 7 solutions as control samples.

**Charred**	**pH**	**20**	**200**	**300**	**400**	**500**	**600**	**700**	**800**	**900**
	**3**	3	3	3	3	3	3	3	3	3
	**7**	1	-	1	-	-	-	1	-	-
	**12**	3	3	3	3	3	3	3	3	3
**Combusted**	**pH**	**20**	**200**	**300**	**400**	**500**	**600**	**700**	**800**	**900**
	**3**	-	3	3	3	3	3	3	3	3
	**7**	-	-	1	-	-	-	1	-	-
	**12**	-	3	3	3	3	3	3	3	3
							**Total unheated**			**7**
							**Total charred**			**50**
							**Total combusted**			**50**
							**Full total**			**107**

Supplementary Information 1: figures

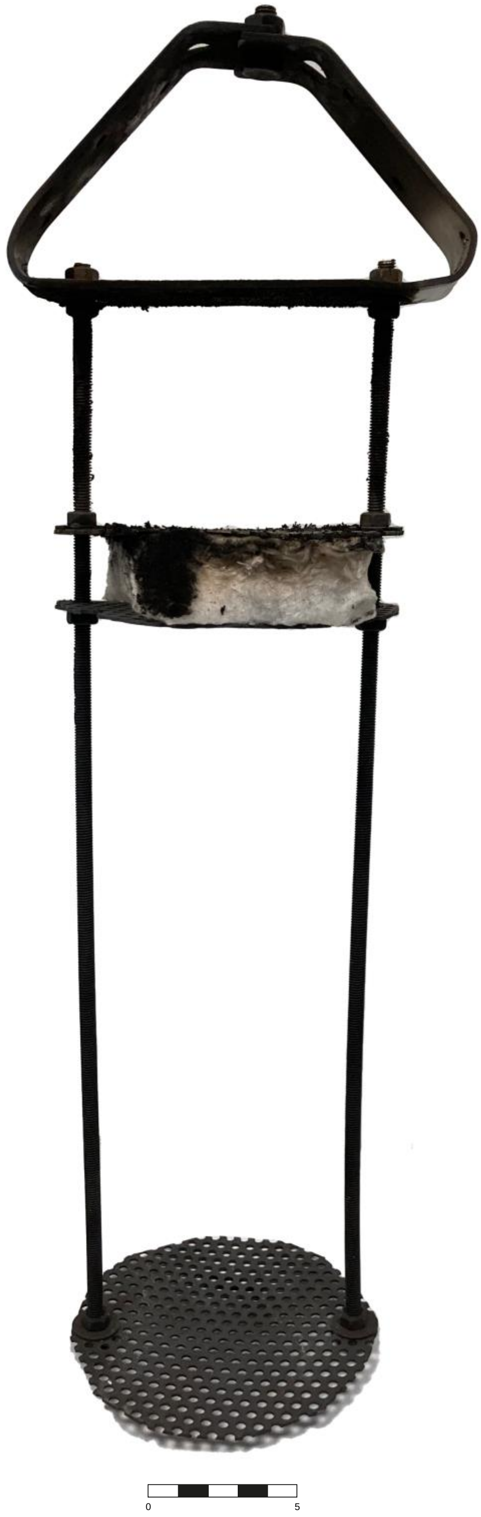

Figure SI.1: The custom oven rig used with the Carbolite GVA 12/300 tube furnace to produce the combusted bone samples. The rig allowed the samples to be placed in the middle of the furnace tube. The mesh platform allowed for sufficient airflow, while additional insulation at the top kept the temperature in the furnace more constant despite the airflow.

SI 2: Results

All reference data for untreated heated bone derives from previous studies by the author: charred bone [SI.4], combusted bone [SI.5].

SI 2.1 Physical analysis

SI 2.1.1 Colour

Colour change results are presented in Fig. SI.2a, b, and c and as Munsell codes in Table SI.2a, b, and c. While Munsell codes are a good way to describe the colour of heated bones in a systematic and reproducible way, the Munsell colour chart turned out not to adequately capture the colour variation of heated bone exposed to pH solutions. This was the case for the unheated samples, as well as those charred to 400 and 700 °C and samples combusted at 600 °C.

Exposure to pH 3 solutions results in darker colours. This is the case for bone charred to 300 and 400 °C. At higher temperatures no colour change can be detected because all samples are black. For combusted bone exposure to pH 3 results in darker colours for samples heated to 200 – 600 °C. At 700 – 900 °C no colour change can be detected because all samples are stark white. The colour of unheated bone appears not to be affected by exposure to pH 3 conditions.

Exposure to pH 12 solutions results in lighter colours in both charred and combusted bone, as well as in unheated bone. Charred bone samples display a lighter colour at temperatures from 200 to 700 °C, while for combusted bone lighter colours appear from 200 to 500 °C.

Exposure to pH 7 solutions has no effect on the two available combusted bone samples (300 and 700 °C), while it results in lighter colours in the two available charred bone samples and unheated bone sample (20, 300, 700 °C).

SI 2.1.2 Fragmentation

Fragmentation as a result of exposure to different pH conditions occurred for 52% (n = 56) of the samples (n = 107), and in equal proportions for charred and combusted bone (Table SI.3a, b, c). For both charred and combusted bone (both n = 50) 56% of the samples were fragmented after incubation. Notably, no fragmentation occurred for the unheated bone samples (n = 7). Exposure to pH 3 and pH 12 both resulted in 53% (n = 27) fragmentation of the samples (n = 51). The effect of pH 3 was slightly larger for combusted bone than for charred bone (58% vs. 54%), with the reverse being true for exposure to pH 12 (charred 58% vs. combusted 54%). Of the samples exposed to pH 7 (n= 5) 40% were fragmented (n = 2).

For the different temperature groups the data show that the percentage of fragmentation increases with increasing temperature (200-400 °C: 32%; 500-600 °C: 42%; 700-900 °C: 90%). At 200-400 °C combusted bone is more susceptible to fragmentation than charred bone, particularly when exposed to pH 3 (44% vs. 22%). However, at 500-600 °C, combusted bone is much less susceptible to fragmentation than charred bone, regardless of pH (17% vs. 67%). At 700-900 °C, 100% of the combusted bone samples are fragmented, versus 78% of the charred bone samples. For exposure to pH 7 only samples heated to 300 and 700 °C were tested (n = 4), and among those only those heated to 700 °C showed fragmentation (100%).

Fragmentation was also classified into groups based on number of fragments: 2-4, 5-7, and 8-10 pieces. In the first group (2-4 pieces) more fragments of charred bone are present than for combusted bone. For group two (5-7 pieces) the same number of fragments for charred and

combusted bone is found, with the exception of combusted bone exposed to pH 12, which has double the number of fragments (17% vs 8%). In the third group (8-10 pieces) combusted bone samples prevail over charred bone samples, regardless of pH.

SI 2.1.3 Mass loss

Percentages of mass loss can be found in Fig. SI.3a and b, and in Table SI.2a, b, and c. Results show that exposure to acidic conditions of unheated bone results in much higher mass loss than exposure to neutral and alkaline conditions (15.4% vs. 5.6 and 5.7%).

The highest mass loss for exposure to pH 3 occurs for bones heated to 200 °C (charred: 16.8%, combusted: 19.8%). At 300 °C mass loss is still quite high (charred: 14.9%, combusted: 8.6%). At these temperatures the mass loss is lower than and similar to pH 12 values, respectively, for charred bone, while for combusted bone mass loss for pH 3 is higher for both temperatures. Between 400 and 900 °C mass loss for charred bone exposed to pH 3 is consistently higher than for pH 12, and decreases with increasing temperature (from 9 to 5.4%). Mass loss for combusted bone at these temperatures is also consistently higher for pH 3 than for pH 12, but values fluctuate (between 8.2 and 4.6%).

The highest mass loss for exposure to pH 12 also occurs for bones heated to 200 °C (charred: 20.9%, combusted: 18%), with the mass loss for charred bone exceeding those resulting from pH 3, and the values for combusted bone being slightly lower than those resulting from pH 3. At 300 °C mass loss for charred bone is 15.1% (similar to pH 3) and 7.1% for combusted bone (<pH 3). From 400 °C onwards mass loss values are very low (< 3%) and often dip below zero, suggesting a slight mass increase. The latter occurs more often for charred bone than for combusted bone (at 500-900 °C vs. at 400 + 600 °C).

At 300 °C, mass loss for bone exposed to pH 7 is much lower than for that exposed to pH 3 and pH 12, both for charred and combusted bone (charred: 7.5%, combusted: 2.7%). At 700 °C the effect of pH 7 is similar to that of pH 12 (charred: -5.3%, combusted: 1%).

SI 2.1.4 Thermogravimetric analysis (TGA)

TGA results are presented in Fig. SI.4a and b, and in Table SI.2a, b, and c. The effect of the different pH values will be discussed using the values for the organic content. However, it should be noted that any changes in the organic content will result in changes in the relative inorganic content, and vice versa. For example, a relative increase in organic content suggests loss of inorganic compounds. The data shows that exposure of unheated bone to acidic conditions results in a relative increase in organic content, in relation to unexposed bone (25.5 wt% vs. 24.1 wt%). Exposure to alkaline conditions results in a decrease in the organic content compared to unexposed bone (22.3 wt% vs. 24.1 wt%). Unheated bone exposed to neutral conditions appears to behave like unexposed bone (organic content: 23.8 wt%).

Exposure to pH 3 has the most pronounced effect on bones heated to 200 °C, resulting in a decrease in organic content compared to unexposed bone at the same temperature (charred: 14.3 wt% vs. 24 wt%, combusted: 12.3 wt% vs. 24.9 wt%). At 300 °C there is also a decrease in organic content compared to unexposed bone (charred: 10.4 wt% vs. 18.7 wt%, combusted: 12 wt% vs. 15 wt%). For bone charred to 400 °C there is still a small decrease in organic content, while for combusted bone there is only a small relative increase in organic content compared to unexposed bone at the same temperature. At 500 °C the organic content of charred bone exposed to pH 3 overlaps with that of unexposed bone. From 600 °C onwards there is a relative increase in organic content, which peaks at 900 °C. For combusted bone exposed to pH 3 there is a small relative increase in organic content for

bone heated to 500-600 °C, and values overlap with those of unexposed bone for temperatures of 700-900 °C.

Exposure to pH 12 has the most pronounced effect on bones heated to 200 °C, even more so than pH 3, resulting in a decrease in organic content compared to unexposed bone (charred: 3.3 wt% vs. 24 wt%, combusted: 2.7 wt% vs. 24.9 wt%). At 300 °C the organic content of charred bone is similarly low, while for combusted bone the effect is slightly less intense (8.2 wt%). From 400-600 °C there is still a decrease in organic content for charred bone, but the offset from unexposed bone is now much smaller. For combusted bone exposed to pH 12 the organic content overlaps with that of unexposed bone at temperatures of 400-700 °C, with the exception of 600 °C where there is a relative increase. For charred bone organic content values start to overlap with those of unexposed bone at 700-800 °C. At the far end of the temperature range both charred and combusted bone samples show a minor relative increase in organic content, compared to unexposed bone of the same temperature.

Charred bone exposed to pH 7 appears to behave like charred bone exposed to pH 3. Combusted bone heated to 300 °C shows a decrease in organic content, but much less pronounced than in combusted bone exposed to pH 3 and pH 12. At 700 °C all values overlap, regardless of pH. For all combinations of variables (charred, combusted, pH) the effect of pH exposure decreases with increasing temperature.

SI 2.2 Elemental analysis

SI 2.2.1 X-ray fluorescence (XRF)

The results of the XRF analysis are presented in Fig. SI.5a, b, c, d, and Table SI.2a, b, and c. The effect of pH exposure on the elemental composition of heated bone is described based on the two main elements present in bone mineral: calcium and phosphorous. It should be noted that a comparison with the unexposed reference data could not be made. Therefore, only the pH samples will be evaluated here. The used XRF technique compensates elements it cannot detect (especially C, H, N, O) by a balance parameter. This means that when material is lost there is a relative increase in the remaining components. XRF results are therefore also affected by dissolution of compounds that cannot be directly measured by the technique itself, such as organic material (CH_2O) and carbonate (CO_3). The effect of exposure to pH solutions on the CaO and P_2O_5 content cannot be properly assessed without a comparison with unexposed reference data. However, there are clear indications of loss of organic material and carbonates in the XRF results.

For charred bone, exposure to pH 3 conditions results in lower CaO values than for samples exposed to pH 12 solutions. This effect is strongest at low temperatures (200 - 300 °C) and decreases with increasing temperature as the relative organic fraction increases as the inorganic components are lost. Combusted bone exposed to pH 3 conditions shows a similar trend at low temperatures (200 – 400 °C), after which CaO values start to overlap with those for samples exposed to pH 12 conditions. P_2O_5 values in low temperature charred bone (200 – 300 °C) exposed to pH 3 conditions are also lower than in bone exposed to pH 12 conditions. Values overlap at 400 °C and become higher for pH 3 samples than for pH 12 samples from 500 °C onwards. Combusted bone exposed to pH 3 conditions also has lower P_2O_5 values at 200 °C. Values overlap with those for pH 12 samples at 300 °C, and are higher at 400 to 700 °C. At 800 and 900 °C values for both pH conditions overlap and are very close together.

The data show that CaO and P_2O_5 values are higher for pH 12 samples than for pH 3 samples at low temperatures. In the samples exposed to pH3, there is still a lot of organic material present that

would be lost during exposure to pH 12 conditions. CaO and P_2O_5 are higher in pH 3 samples than in pH 12 samples at higher temperatures (>500 °C) where there is little to no effect of loss of organic content. This relative increase compared to the pH 12 treated samples might be related to loss of carbonates as a result of exposure to pH 3 conditions, as carbonate (CO_3) is only lost at > 700 °C in samples exposed to pH 12. Finally, overlapping values for both pH values indicate similar chemical properties (i.e., mainly bone mineral at high temperatures), as well as no or only a minor effect of pH exposure.

SI 2.3 Molecular analysis

SI 2.3.1 Fourier transform infrared spectroscopy (FTIR)

The results of the FTIR analysis are presented in Table SI.2a, b, and c, Fig. SI.6a and b (SF), Fig. SI.7a and b (C/P ratio), and Fig. SI.8a-q (all spectra) and are discussed in terms of curve shape, splitting factor values (SF), and C/P ratio values. For both heated and unheated bone, increased crystallinity and reduced C/P values are observed as exposure to different pH conditions causes loss of both organic and inorganic compounds, compared to unexposed bone.

Exposure to pH 3 solutions mainly affects the inorganic content of both charred and combusted bone, resulting in reduced CO_3 peaks and increased splitting of the PO_4^{3-} v1 symmetric stretching. In charred bone the shoulder on the PO_4^{3-} v3 peak appears at a much lower temperature (at 400 °C instead of at 900 °C). The changes to the PO_4^{3-} peaks suggest both recrystallisation and some loss of phosphates. Loss of organics can also be observed (amides and aromatic compounds), but to a lesser extent than for samples exposed to pH 12 solutions. The loss of CO_3 also becomes clear from the C/P ratio values, which are always lower for bone exposed to pH 3 solutions than for unexposed bone. The C/P ratio differences between pH 3 and pH 12 samples decrease with increased temperature, with the ratio difference being largest for bone heated to 200 °C. Exposure to pH 3 conditions also has a clear effect on the splitting factor, with higher values compared to unexposed bone. The effect is always more pronounced for bone exposed to pH 3 than for those exposed to pH 12, and for charred bone the difference between the two increases with higher temperatures (400 – 900 °C).

Exposure to pH 12 solutions mainly affects the organic content of charred and combusted bone, resulting in loss of amides and aromatic compounds. Some changes in the inorganic content can also be observed (e.g., reduction of CO_3 peaks and increased splitting of the PO_4^{3-} v1 symmetric stretching), but the effect is less pronounced than for samples exposed to pH 3 solutions. The reduction in CO_3 can also be tracked through the C/P ratio. For bone exposed to pH 12 conditions C/P values are lower than those of unexposed bone for all temperatures, except at 600 – 800 °C for charred bone and at 500 – 700 °C for combusted bone. C/P values for bone exposed to pH 12 are always higher than those for bone exposed to pH 3. It should be noted that the reduction of CO_3 peaks likely relates both to loss of carbonates and to loss of organic compounds, with which these peaks overlap. There is also an increase in crystallinity, as reflected by the higher SF values as compared to unexposed heated bone. However, this effect is not as strong as for exposure to pH 3 solutions.

Exposure to pH 7 solutions also results in a decrease in organic compounds and CO_3, as well as increased splitting of the PO_4^{3-} symmetric stretching and increased SF values. However, the effect is generally less pronounced than for the other two pH values.

SI 2.3.2 Pyrolysis gas-chromatography mass spectrometry (Py-GCMS)

The results of the Py-GCMS analysis are grouped in eight different compound types and presented in Table SI.4a, b, and c. The identified compound types are monocyclic aromatic hydrocarbons (MAH), methylene chain compounds (MCC), N-containing compounds (NCOMP), polycyclic aromatic hydrocarbons (PAH), phenols (PHEN), tert-butylphenols (TERT), triterpenoids (TRITERP), and unidentified (OTHER). Due to the difference in technique and resolution (Py-GCMS vs. pyMS and DTMS), a comparison with the heated bone MS reference data is not warranted. Py-GCMS results show that exposure to all pH values has an effect on the organic molecular composition of heated bone. Unheated bone is also affected by pH exposure, but to a lesser extent than heated bone.

Exposure to pH 3 solutions results in dissolution and loss of MAHs (e.g., benzene, toluene, styrene) for both charred and combusted bone, and for all analysed temperatures (20 °C, 200-500 °C). The effect is most noticeable for bone heated to 200 °C and decreases with increasing temperature. MCCs (e.g., alkyl-nitrile, amide, alkanes) and PAHs (e.g., indene, naphthalene, fluorene) are affected by exposure to pH 3 in the low temperature samples, while they are affected by exposure to pH 12 in the medium temperature samples. In charred bone MCCs leach out at 200 and 300 °C, while PAHs leach out for samples heated to 200-400 °C. In combusted bone this trend is reversed, with MCCs leaching out for samples heated to 200-400 °C and PAHs leaching out for 200 and 300 °C. Finally, TERTs also react to exposure to both pH values. In charred bone TERTs dissolve in pH 3 solutions for samples heated to 200-400 °C, while in combusted bone they dissolve in pH 3 for samples heated to 200, 400, and 500 °C. TERT values are univariably low and the same for both pH values in unheated bone.

Exposure to pH 12 solutions results in dissolution and loss of NCOMPs (e.g., pyrrole, pyridine, dipeptides), PHENs (e.g., phenol), TRITERPs (e.g., cholestadiene, cholestadienol), and some unidentified compounds (OTHER) for both charred and combusted bone, as well as for unheated bone. For NCOMPs, the largest category, many compounds are completely lost as a result of exposure to pH 12, especially for bone heated to 200 °C (e.g., diketodipyrrole). The effect decreases with increasing temperature, alongside the overall decrease in NCOMPs as a result of heating. In both charred and combusted bone PHENs leach out as a result of exposure to pH 12 solutions, sometimes completely (e.g., at 200 °C). TRITERPs, present at low temperatures (20 °C, charred 200-300 °C, combusted 200 °C), are also completely lost after exposure to pH 12. While being affected by pH 3 at low temperatures, MCCs and PAHs leach out in pH 12 at higher temperatures (charred bone: MCC at 400-500 °C, PAH at 500 °C; combusted: MCC at 500 °C, PAH at 400-500 °C). Finally, TERTs leach out in pH 12 solutions for bone charred to 500 °C and for bone combusted to 300 °C.

For exposure to pH 7 solutions only 3 samples were available for Py-GCMS analysis: unheated bone, charred 300 °C, and combusted 300 °C. For unheated bone the effect of pH 7 is somewhere in between that of pH 3 and pH 12, i.e., some loss of compounds. However, at 300 °C there is no clear pattern and sometimes compound values are lower after exposure to pH 7 than for exposure to the other two pH values.

SI 2.4 Structural analysis

SI 2.4.1 X-ray diffraction (XRD)

The results of the XRD analysis are presented in Fig. SI.9a and b (CI), Fig. SI.10a-k (all diffractograms), and in Table SI.2a, b, and c. Measurements were performed on unheated bone, as well as on bone heated to 500°C and higher as this is where changes in crystallinity start to occur during heating. Results show that exposure to all pH values results in increased crystallinity, with the effect of pH 3

being more prominent. Exposure to pH solutions did not have an effect on the crystallinity of unheated bone, where all samples showed CI values of 0.00 or 0.01.

Exposure to pH 3 results in the largest increase in crystallinity, for all charred bone samples and for combusted bone heated between 500 and 600°C. In both cases the effect increases with increasing temperature. The effect is also visible in the diffractograms as sharper, more pronounced peaks over the full 2θ range for samples exposed to pH 3. The exception to this trend is the combusted bone samples heated to 700°C and above. For these samples there is still an increase in crystallinity, but here the effect of pH 12 is more pronounced. Exposure to both pH values results in a decrease in crystallinity for combusted bone heated to 900°C. This may be related to the process of dehydroxylation of the bone mineral, which is known to start around that temperature in combusted bone (Tonsuaadu et al., 2012).

Exposure to pH 12 results in an increase in crystallinity in charred bone that is less severe compared to exposure to pH 3. For 600 and 700°C CI values are higher, but still very similar to those of unexposed charred bone. The increase in crystallinity becomes a bit bigger for bone charred to 800 and 900°C. For combusted bone heated to 500°C and exposed to pH 12 the CI values overlap with those of samples exposed to pH 3, but are much higher than those of unexposed bone. For combusted bone heated to 600°C the increase in crystallinity is similar, but lower than for the samples exposed to pH 3. At 700 and 800°C, the trend reverses and samples exposed to pH 12 start showing the highest crystallinity increase. At 900°C another switch occurs, now resulting in a decrease in crystallinity, which is less defined for pH 12 than for pH 3. This may be related to dehydroxylation reactions that are known to occur in combusted bone at this temperature [SI.6].

Exposure to pH 7 appears most similar to exposure to pH 12. However, it should be noted that only two pH 7 samples were available for XRD analysis (charred + combusted bone 700°C).

References

SI.4 Reidsma, F. H., van Hoesel, A., van Os, B. J. H., Megens, L. & Braadbaart, F. Charred bone: physical and chemical changes during laboratory simulated heating under reducing conditions and its relevance for the study of fire use in archaeology. *J. Archaeol. Sci. Reports* **10,** 282–292, doi: 10.1016/j.jasrep.2016.10.001 (2016).

SI.5 van Hoesel, A., Reidsma, F. H., van Os, B. J. H., Megens, L. & Braadbaart, F. Combusted bone: physical and chemical changes of bone during laboratory simulated heating under oxidising conditions and their relevance for the study of ancient fire use. *J. Archaeol. Sci. Reports* **28,** doi: 10.1016/j.jasrep.2019.102033 (2019).

SI.6 Tõnsuaadu, K., Gross, K. A., Pluduma, L. & Veiderma, M. A review on the thermal stability of calcium apatites. *J. Therm. Anal. Calorim.* **110,** 647–659, doi: 10.1007/s10973-011-1877-y (2012).

Supplementary Information 2: tables

Table SI.2a: Overview table showing the numeric results for the samples exposed to pH 3 conditions, per analytical technique. For the XRF results the TGA-corrected values are displayed. A dash (-) indicates that particular samples were not analysed for that technique. This table is also presented in the main text as Table 2a.

HC	Temp	Colour	Mass Loss	TGA			XRF			FTIR		XRD
				Water	Organic	Ash	CaOcorr	P2O5corr	Ca/P	SF	C/P	CI
	°C	Munsell	wt%	wt%	wt%	wt%	wt%	wt%	-	-	-	-
unheated	20	5Y8/1	15.43	6.91	25.46	65.82	37.00	28.04	1.32	4.32	0.27	0.01
charred	200	2.5Y8/4	16.80	4.00	14.25	79.71	43.55	35.57	1.22	4.35	0.19	-
charred	300	5YR3/5	14.87	3.59	10.37	84.02	46.53	36.93	1.26	4.61	0.15	-
charred	400	5YR2/2	9.02	3.37	8.92	85.75	46.53	38.63	1.20	4.90	0.12	-
charred	500	5YR2/2	7.92	4.27	8.55	85.66	45.74	39.23	1.16	5.18	0.12	0.07
charred	600	5YR2/1	6.51	4.21	7.89	86.40	45.35	40.49	1.12	5.32	0.11	0.15
charred	700	7.5YR2/0	7.21	4.00	6.86	87.75	47.71	39.42	1.21	5.57	0.10	0.22
charred	800	7.5YR2/0	5.39	2.89	5.57	90.02	48.84	40.55	1.20	6.09	0.07	0.38
charred	900	7.5YR2/0	5.39	2.14	3.54	93.42	50.89	41.89	1.21	7.15	0.03	0.72
combusted	200	10YR7/4	19.84	4.81	12.33	81.18	42.20	38.36	1.10	4.61	0.16	-
combusted	300	5YR3/4	8.59	5.04	11.95	81.24	41.58	39.09	1.06	4.50	0.18	-
combusted	400	5YR4/1	6.21	3.99	5.08	89.27	46.92	41.69	1.13	4.90	0.12	-
combusted	500	5YR5/2	7.05	3.49	3.80	91.15	49.02	41.52	1.18	5.12	0.10	0.21
combusted	600	7.5YR4/0	4.62	2.43	1.97	94.40	51.62	42.15	1.22	6.02	0.07	0.63
combusted	700	5YR8/1	8.15	0.62	0.29	98.76	54.23	43.89	1.24	6.67	0.04	1.18
combusted	800	5YR8/1	7.33	0.58	0.22	98.89	53.61	44.61	1.20	6.44	0.04	1.17
combusted	900	5YR8/1	7.09	0.47	0.22	98.95	53.34	44.89	1.19	5.83	0.04	1.12

Table SI.2b: Overview table showing the numeric results for the samples exposed to pH 12 conditions, per analytical technique. For the XRF results the TGA-corrected values are displayed. A dash (-) indicates that particular samples were not analysed for that technique. This table is also presented in the main text as Table 2b.

HC	Temp	Colour	Mass Loss	TGA			XRF			FTIR		XRD
				Water	Organic	Ash	CaOcorr	P2O5corr	Ca/P	SF	C/P	CI
	°C	Munsell	wt%	wt%	wt%	wt%	wt%	wt%	-	-	-	-
unheated	20	5Y8/1	5.74	6.75	22.28	68.49	38.50	28.87	1.33	3.99	0.28	0.00
charred	200	5Y8/1	20.86	4.14	3.33	89.62	50.20	38.42	1.31	4.28	0.16	-
charred	300	10YR6/4	15.07	4.26	3.29	89.62	50.24	38.40	1.31	4.34	0.15	-
charred	400	5YR3/2	2.61	4.66	6.30	86.15	47.06	38.03	1.24	4.15	0.18	-
charred	500	5YR2/1	-2.73	4.90	6.76	85.44	46.61	37.68	1.24	4.31	0.17	0.06
charred	600	5YR2/1	-4.91	4.97	6.81	85.36	46.67	37.55	1.24	4.27	0.18	0.06
charred	700	7.5YR2/0	-2.03	5.19	6.14	85.67	47.75	36.92	1.29	4.64	0.17	0.06
charred	800	7.5YR2/0	-3.09	3.79	4.35	89.24	49.62	38.52	1.29	5.01	0.13	0.17
charred	900	7.5YR2/0	-1.1	2.27	2.78	93.17	51.31	41.14	1.25	5.39	0.07	0.57
combusted	200	5Y8/1	18.02	4.84	2.68	89.55	46.98	41.54	1.13	4.12	0.18	-
combusted	300	5YR4/3	7.08	5.42	8.20	83.51	43.72	38.75	1.13	4.13	0.21	-
combusted	400	5YR5/2	-2.25	4.57	3.82	88.53	48.03	39.38	1.22	4.10	0.19	-
combusted	500	5YR5/2	1.61	4.39	3.20	89.48	49.11	39.27	1.25	4.22	0.17	0.04
combusted	600	7.5YR4/0	-0.95	2.51	1.67	93.63	51.41	41.40	1.24	5.19	0.10	0.49
combusted	700	5YR8/1	1.03	0.87	0.66	97.81	53.96	43.14	1.25	6.53	0.06	1.23
combusted	800	5YR8/1	1.63	0.71	0.57	98.24	53.14	44.47	1.19	5.99	0.06	1.21
combusted	900	5YR8/1	0.63	0.61	0.55	98.20	52.92	44.59	1.19	5.52	0.05	1.15

Table SI.2c: Overview table showing the numeric results for the samples exposed to pH 7 conditions, per analytical technique. For the XRF results the TGA-corrected values are displayed. A dash (-) indicates that particular samples were not analysed for that technique. This table is also presented in the main text as Table 2c.

HC	Temp	Colour	Mass Loss	TGA			XRF			FTIR		XRD
				Water	Organic	Ash	CaOcorr	P2O5corr	Ca/P	SF	C/P	CI
	°C	Munsell	wt%	wt%	wt%	wt%	wt%	wt%	-	-	-	-
unheated	20	5Y8/1	5.64	7.32	23.78	66.80	35.72	30.57	1.17	4.01	0.26	0.00
charred	300	7.5YR6/4	7.49	4.85	10.06	82.81	44.27	38.02	1.16	4.12	0.19	-
charred	700	7.5YR2/0	-5.29	5.04	6.61	86.04	45.80	39.68	1.15	4.78	0.15	0.07
combusted	300	5YR4/2	2.67	5.38	13.86	78.45	41.98	35.96	1.17	4.21	0.20	-
combusted	700	5YR8/1	0.95	0.66	0.62	98.21	52.85	44.71	1.18	6.64	0.05	1.27

Table SI.3a: Overview of the amount of fragmentation that occurred as a result of pH exposure.

A

	**Samples (n)**	**Fragmented (n)**	**Fragmented (%)**
**Unheated**	**7**	**0**	**0.0**
Unh pH 3	3	0	0.0
Unh pH 7	1	0	0.0
Unh pH 12	3	0	0.0
**Charred**	**50**	**28**	**56.0**
Char pH 3	24	13	54.2
Char pH 7	2	1	50.0
Char pH 12	24	14	58.3
**Combusted**	**50**	**28**	**56.0**
Comb pH 3	24	14	58.0
Comb pH 7	2	1	50.0
Comb pH 12	24	13	54.2
**Total**	**107**	**56**	**52.3**
Total pH 3	51	27	52.9
Total pH 7	5	2	40.0
Total pH 12	51	27	52.9

Table SI.3b: Overview of the amount of fragmentation grouped per temperature category
Table SI.3c: Overview of the amount of fragmentation grouped per number of fragments

B

	Samples (n)	Fragmented (n)	Fragmented (%)
**200 – 400 °C**	**38**	**12**	**31.6**
Char pH 3	9	2	22.2
Char pH 7	1	0	0.0
Char pH 12	9	3	33.3
Comb pH 3	9	4	44.4
Comb pH 7	1	0	0.0
Comb pH 12	9	3	33.3
**500 – 600 °C**	**24**	**10**	**41.7**
Char pH 3	6	4	66.7
Char pH 7	0	0	0.0
Char pH 12	6	4	66.7
Comb pH 3	6	1	16.7
Comb pH 7	0	0	0.0
Comb pH 12	6	1	16.7
**700 – 900 °C**	**38**	**34**	**89.5**
Char pH 3	9	7	77.8
Char pH 7	1	1	100.0
Char pH 12	9	7	77.8
Comb pH 3	9	9	100.0
Comb pH 7	1	1	100.0
Comb pH 12	9	9	100.0

C

	Samples (n)	Fragmented (n)	Fragmented (%)
**2 – 4 fragm.**	**56**	**40**	**71.4**
Char pH 3	24	10	41.7
Char pH 7	2	1	50.0
Char pH 12	24	12	50.0
Comb pH 3	24	9	37.5
Comb pH 7	2	1	50.0
Comb pH 12	24	7	29.2
**5 – 7 fragm.**	**56**	**10**	**17.9**
Char pH 3	24	2	8.3
Char pH 7	2	0	0.0
Char pH 12	24	2	8.3
Comb pH 3	24	2	8.3
Comb pH 7	2	0	0.0
Comb pH 12	24	4	16.7
**8 – 10 fragm.**	**56**	**6**	**10.7**
Char pH 3	24	1	4.2
Char pH 7	2	0	0.0
Char pH 12	24	0	0.0
Comb pH 3	24	3	12.5
Comb pH 7	2	0	0.0
Comb pH 12	24	2	8.3

Table SI.4a: Overview of the percentages of different compound classes present in samples exposed to pH 3 conditions. For a breakdown of the different classes see section SI 2.3.2.

A

HC	Class	Temperature				
		20 °C	200 °C	300 °C	400 °C	500 °C
charred	MAH	9.40	12.70	27.57	37.26	35.99
charred	MCC	0.45	5.09	1.67	19.00	52.36
charred	NCOMP	87.48	81.07	66.77	37.65	1.55
charred	PAH	0.48	0.22	1.02	1.95	0.45
charred	PHEN	1.86	0.59	2.71	3.30	0
charred	TERT	0.01	0.09	0.21	0.84	9.66
charred	TRITERP	0.07	0.23	0.06	0	0
charred	OTHER	0.25	0.02	0	0	0
combusted	MAH	9.40	13.22	34.37	41.54	14.93
combusted	MCC	0.45	1.35	2.88	39.72	71.01
combusted	NCOMP	87.48	83.01	57.27	9.14	2.61
combusted	PAH	0.48	0.57	1.29	2.08	0.81
combusted	PHEN	1.86	1.27	2.62	0	0
combusted	TERT	0.01	0.45	1.57	7.51	10.63
combusted	TRITERP	0.07	0.10	0	0	0
combusted	OTHER	0.25	0.01	0	0	0

Table SI.4b + c: Overview of the percentages of different compound classes present in samples exposed to pH 12 (B) and pH 7 (C) conditions. For a breakdown of the different classes see section SI 2.3.2.

B

HC	Class	Temperature				
		20 °C	200 °C	300 °C	400 °C	500 °C
charred	MAH	16.1	53.33	54.16	52.52	51.62
charred	MCC	0.34	9.74	8.47	6.77	39.28
charred	NCOMP	82.15	30.88	32.60	36.51	0.35
charred	PAH	0.34	4.94	3.59	3.07	0
charred	PHEN	0.94	0	0.06	0.24	0
charred	TERT	0.02	1.12	1.12	0.89	8.75
charred	TRITERP	0.04	0	0	0	0
charred	OTHER	0.07	0	0	0	0
combusted	MAH	16.10	67.02	49.42	42.34	30.42
combusted	MCC	0.34	11.56	5.39	44.86	57.80
combusted	NCOMP	82.15	19.04	41.06	0	0.62
combusted	PAH	0.34	1.46	2.64	0	0
combusted	PHEN	0.94	0	0.73	0	0
combusted	TERT	0.02	0.91	0.74	12.80	11.17
combusted	TRITERP	0.04	0	0	0	0
combusted	OTHER	0.07	0	0	0	0

C

HC	Class	Temperature	
		20 °C	300 °C
charred	MAH	11.97	27.86
charred	MCC	0.63	1.09
charred	NCOMP	84.13	68.39
charred	PAH	0.57	1.11
charred	PHEN	2.42	1.32
charred	TERT	0.05	0.21
charred	TRITERP	0.06	0.01
charred	OTHER	0.19	0
combusted	MAH	11.97	30.06
combusted	MCC	0.63	9.22
combusted	NCOMP	84.13	57.69
combusted	PAH	0.57	1.16
combusted	PHEN	2.42	1.61
combusted	TERT	0.05	0.26
combusted	TRITERP	0.06	0
combusted	OTHER	0.19	0

Supplementary Information 2: figures

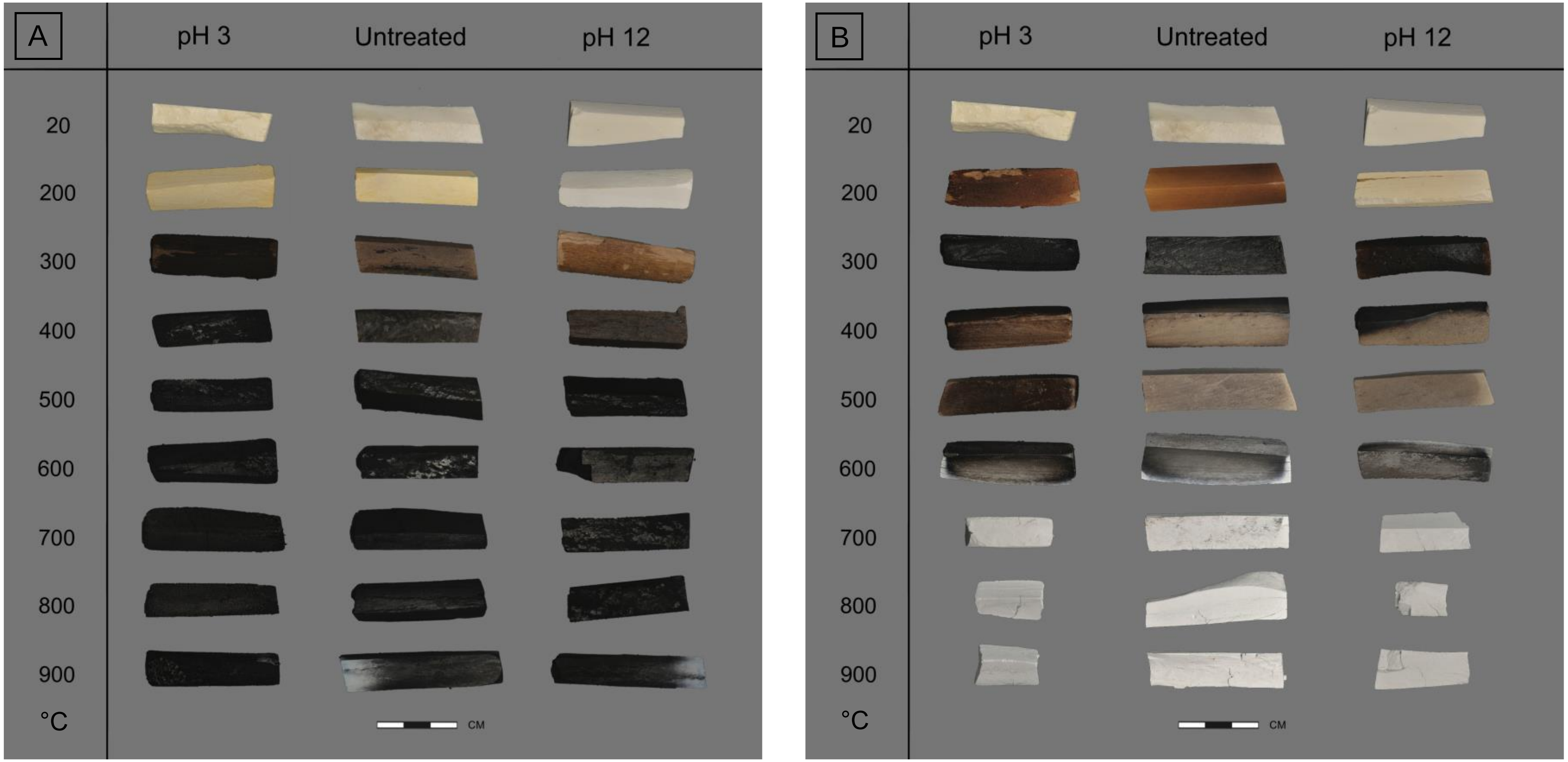

Figure SI.2a, b, c: Overview of the variation in colour for charred (A) and combusted (B) bone exposed to pH 3 and pH 12 conditions, as well as control samples exposed to pH 7 conditions (C). SI.2a and b are duplicates of Fig. 1a and b presented in the main text.

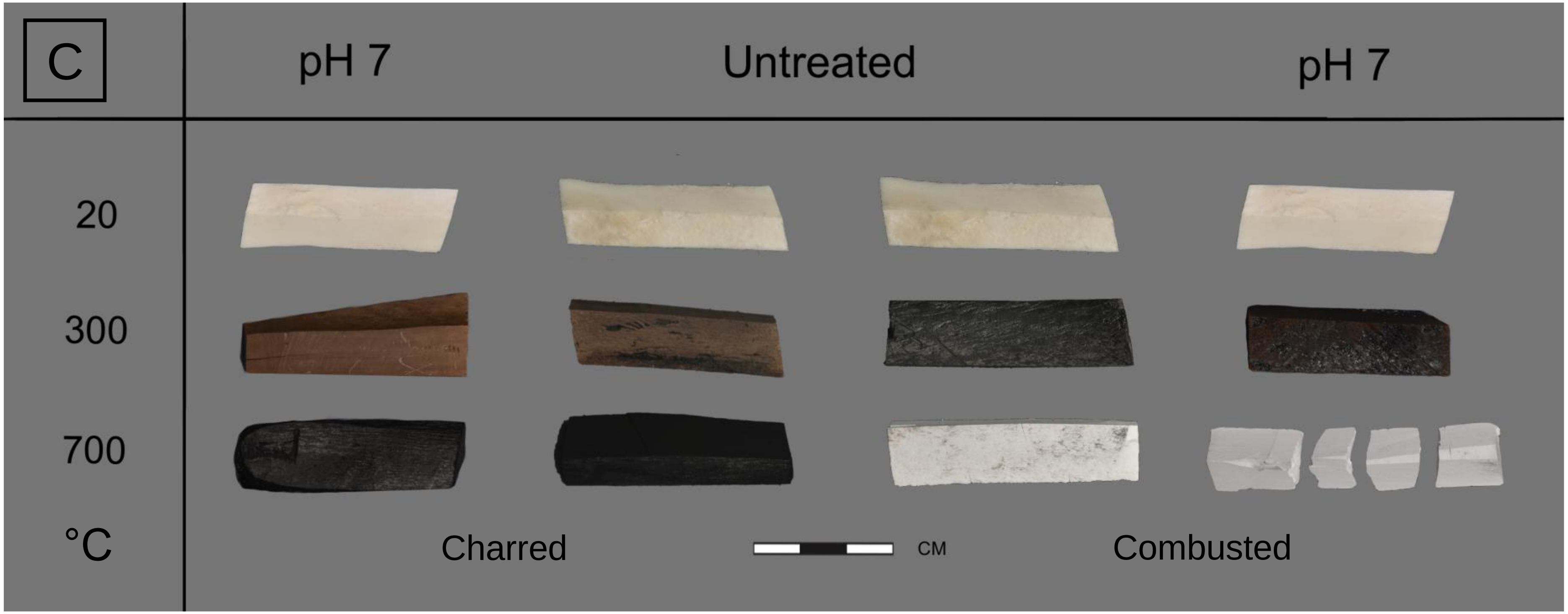
C
pH 7
Untreated
pH 7
20
300
700
°C
Charred
CM
Combusted

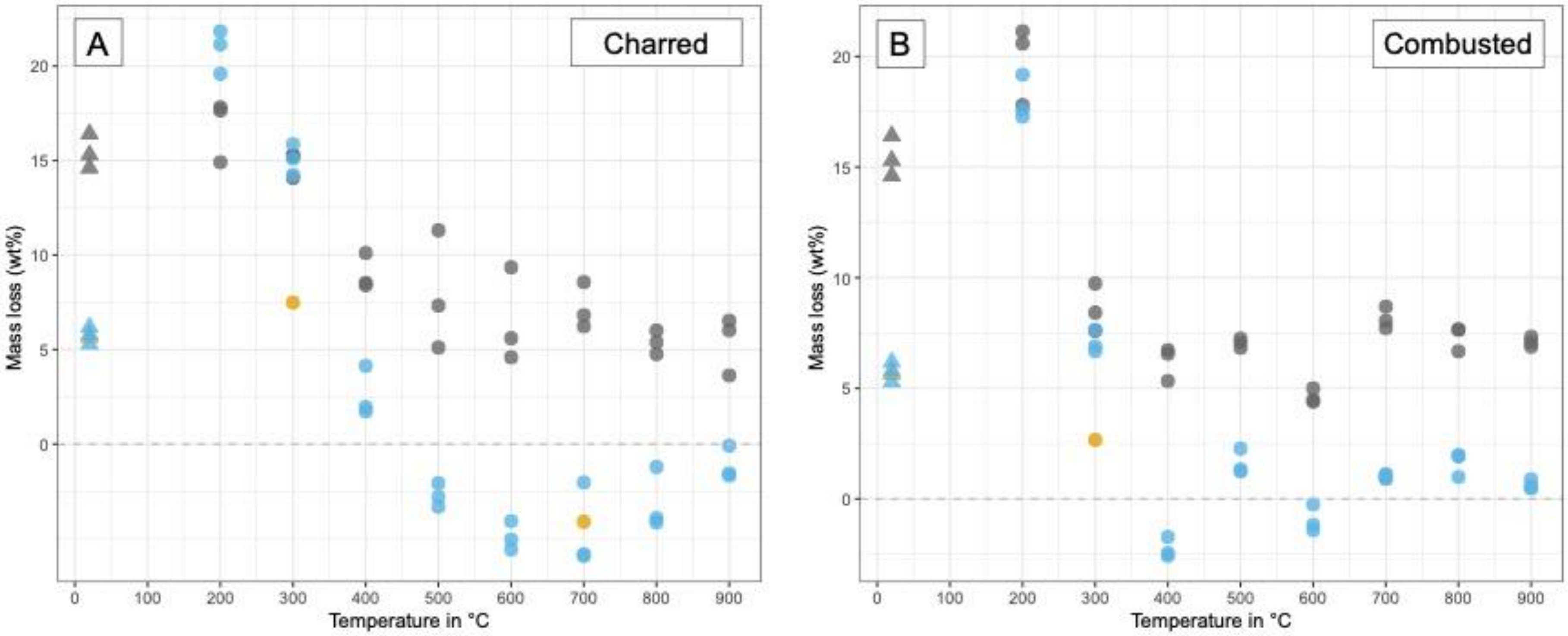

Figure SI.3: Graphs showing the mass loss (wt%) as a result of pH exposure for charred bone (A) and combusted bone (B). Triangles = unheated bone, circles = heated bone, grey = pH 3 samples, yellow = pH 7 samples, blue = pH 12 samples, dashed horizontal line = zero mass loss. These graphs are duplicates of Fig. 2a and b presented in the main text.

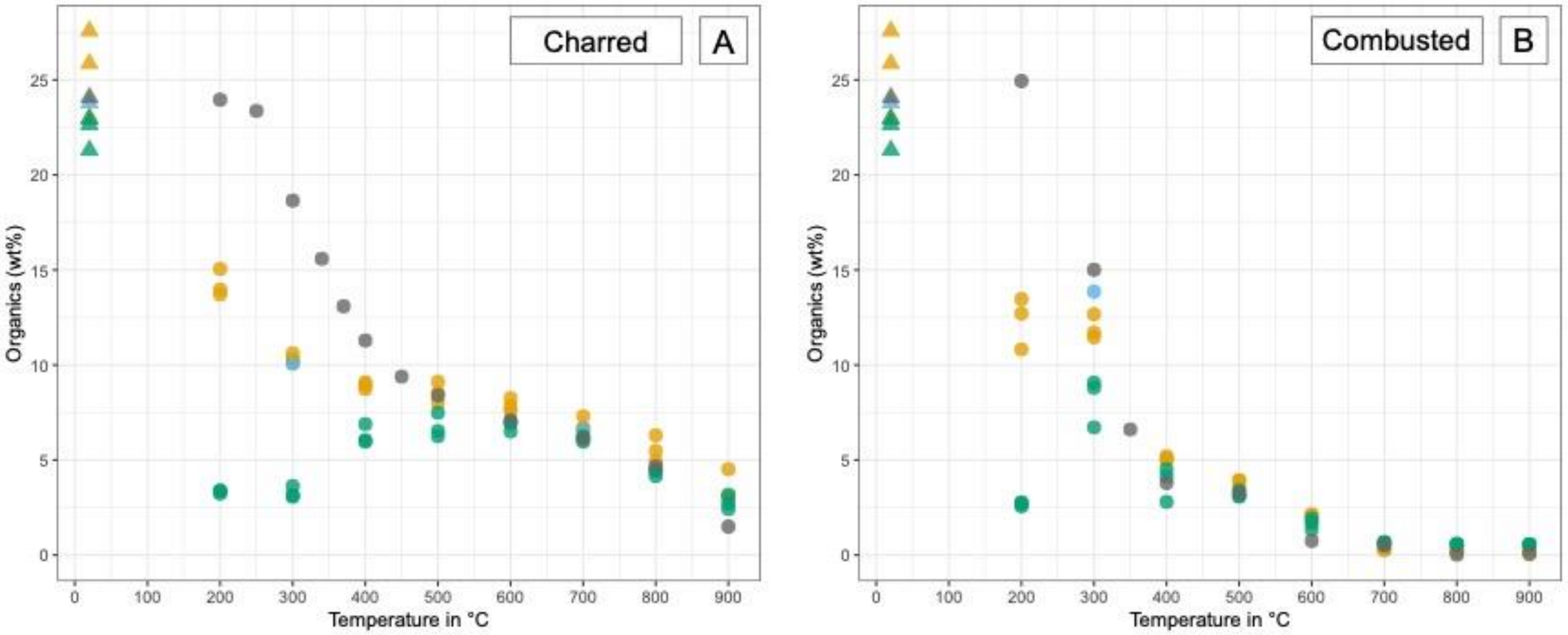

Figure SI.4: Graphs showing the variation in organic content (wt%) in charred (A) and combusted (B) bone, as measured by TGA, as a result of pH exposure. Triangles = unheated bone, circles = heated bone, grey = untreated samples, yellow = pH 3 samples, blue = pH 7 samples, green = pH 12 samples. These graphs are duplicates of Fig. 5a and b presented in the main text.

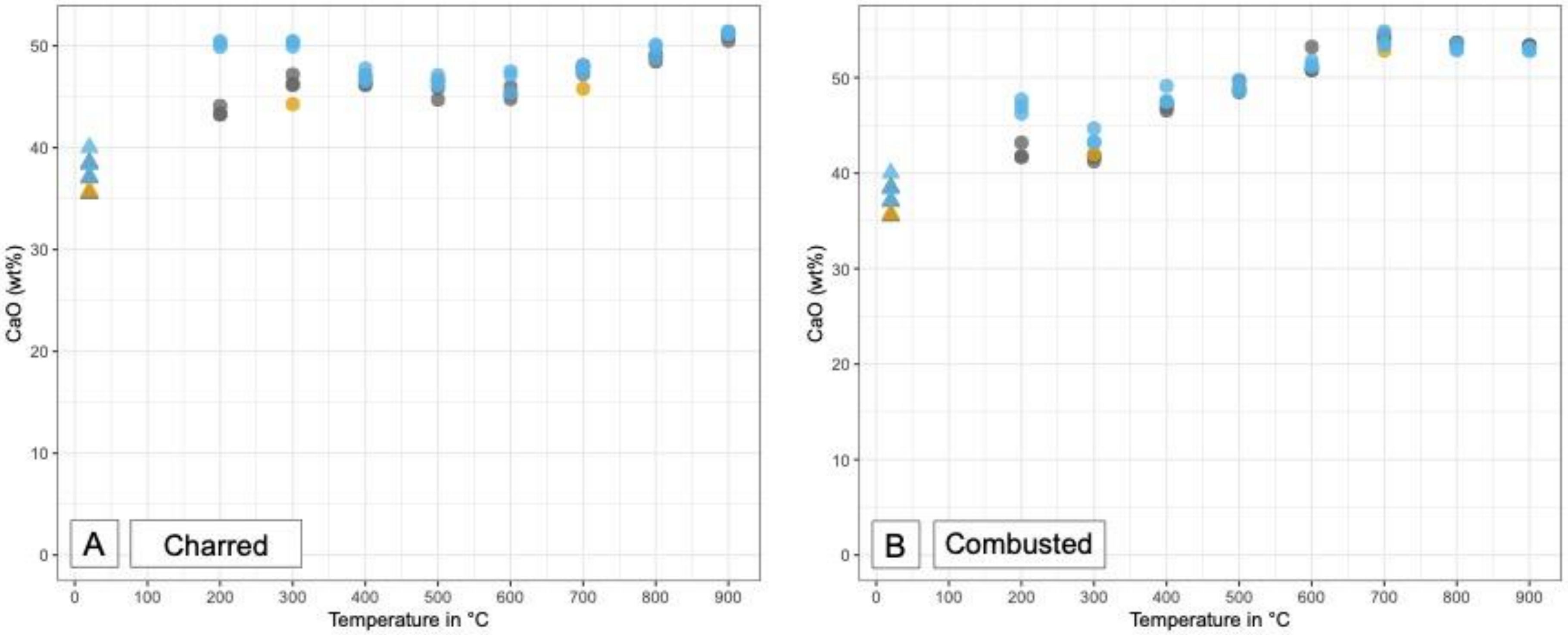

Figure SI.5: Graphs showing the variation in CaO content (wt%, TGA-corrected values) as a result of pH exposure for charred bone (A) and combusted bone (B). Triangles = unheated bone, circles = heated bone, grey = pH 3 samples, yellow = pH 7 samples, blue = pH 12 samples.

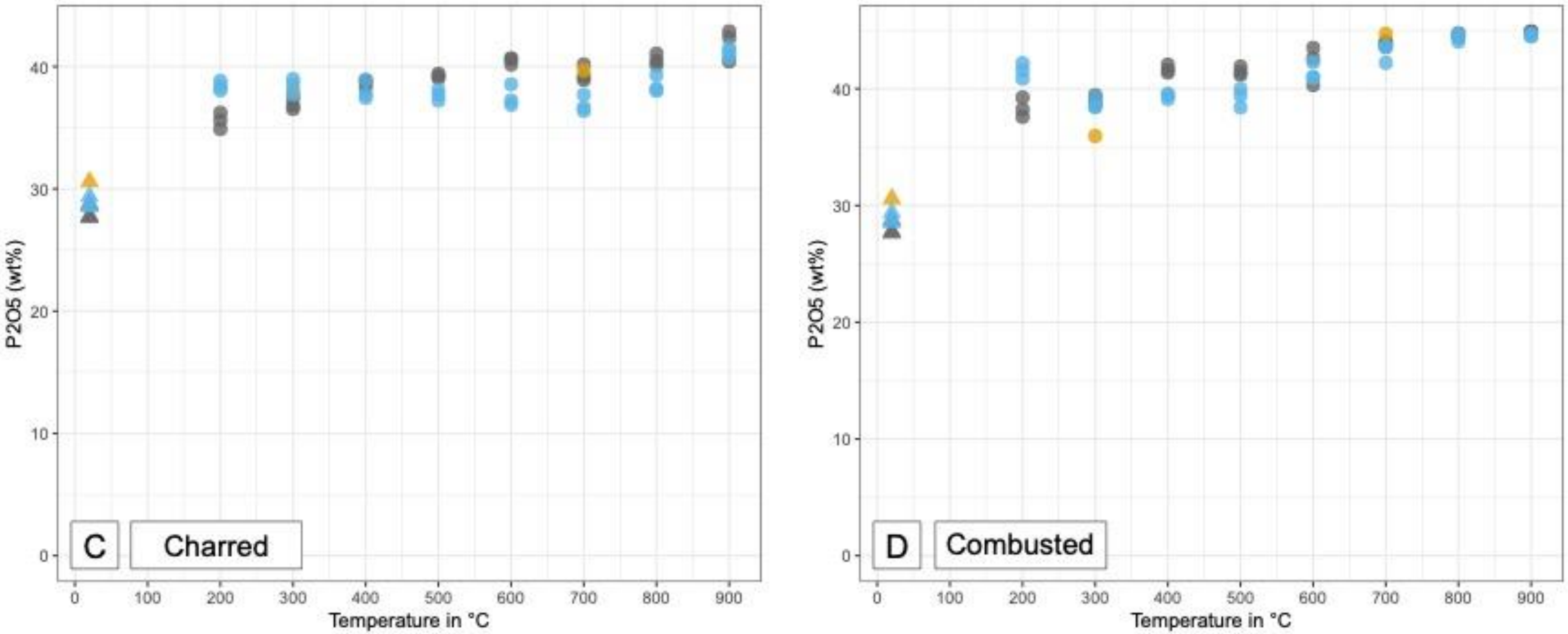

Figure SI.5: Graphs showing the variation in P_2O_5 content (wt%, TGA-corrected values) as a result of pH exposure for charred bone (C) and combusted bone (D). Triangles = unheated bone, circles = heated bone, grey = pH 3 samples, yellow = pH 7 samples, blue = pH 12 samples.

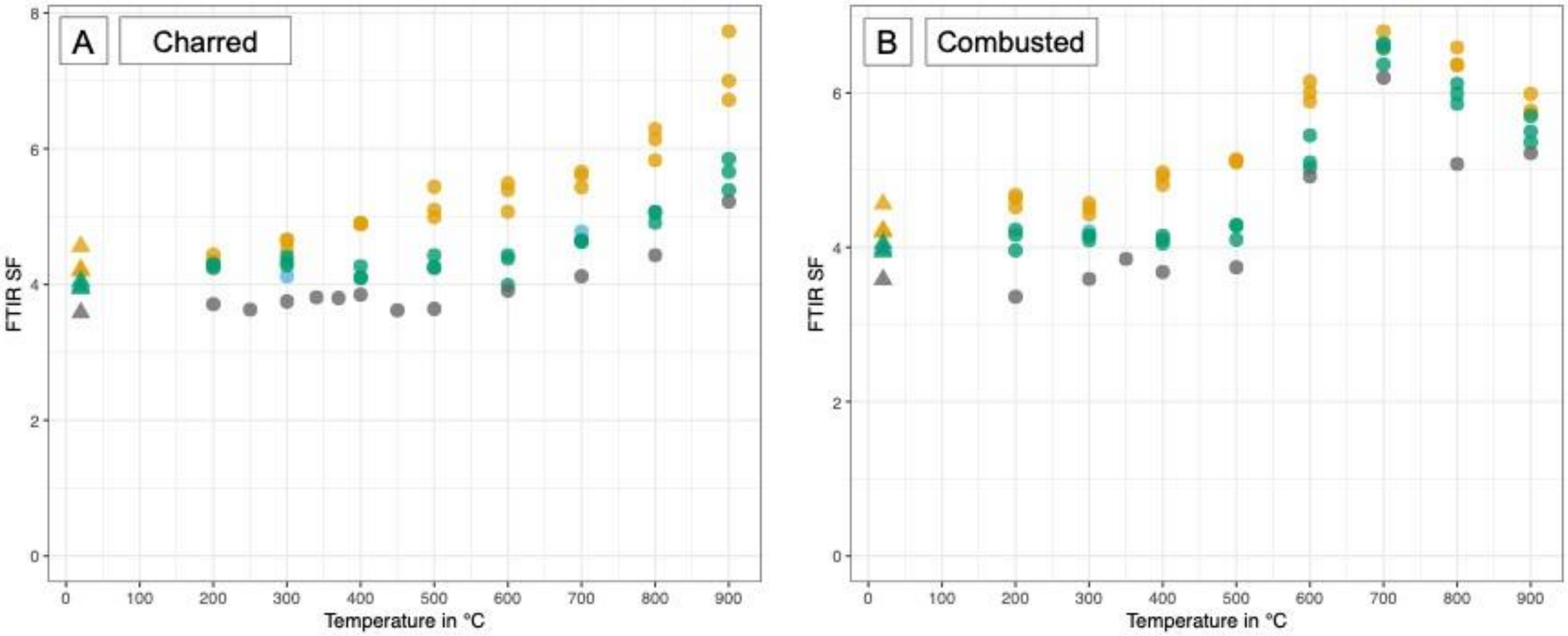

Figure SI.6: Graphs showing the variation in FTIR Splitting Factor values in charred (A) and combusted (B) bone as a result of pH exposure. Triangles = unheated bone, circles = heated bone, grey = untreated samples, yellow = pH 3 samples, blue = pH 7 samples, green = pH 12 samples.

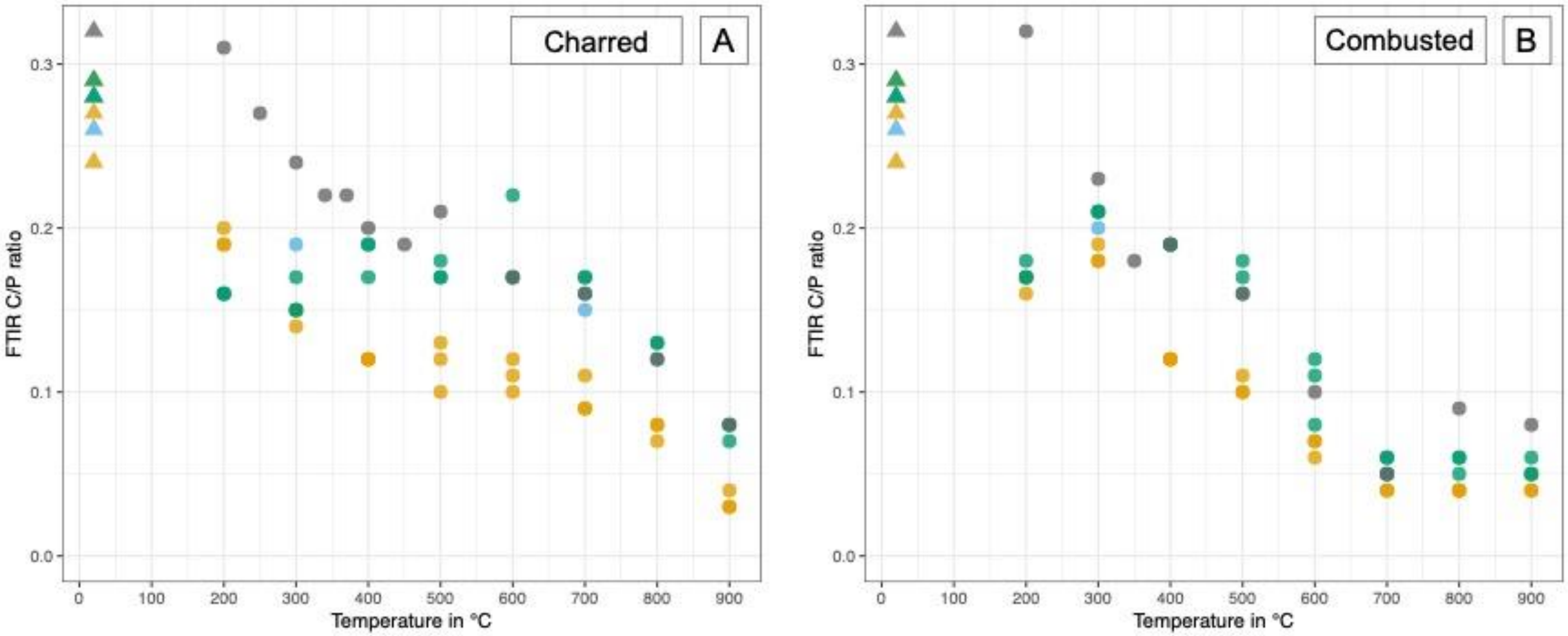

Figure SI.7: Graphs showing the variation in FTIR C/P ratio values in charred (A) and combusted (B) bone as a result of pH exposure. Triangles = unheated bone, circles = heated bone, grey = untreated samples, yellow = pH 3 samples, blue = pH 7 samples, green = pH 12 samples.

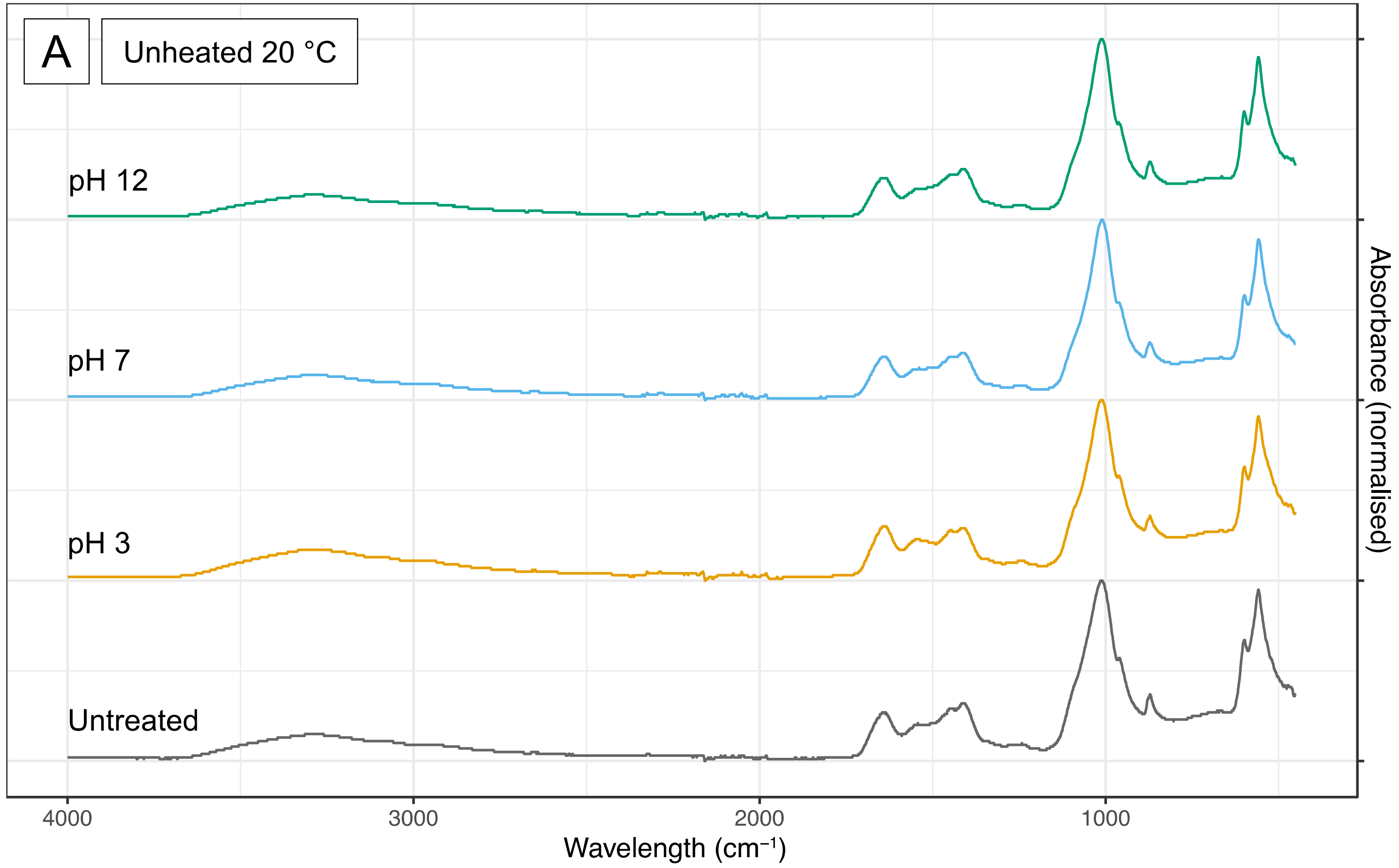

Figure SI.8a-q: FTIR spectra for all temperature-pH combinations, compared to their untreated equivalent. Unheated (A) above, charred and combusted bone (B-Q) below, with charred bone in the left column and combusted bone in the right column. Grey = untreated, yellow = pH 3, blue = pH 7, green = pH 12. For peak identifications see Fig. 4 in the main text. SI.8d, e, l, and m are duplicates of Fig. 4a, b, and c presented in the main text.

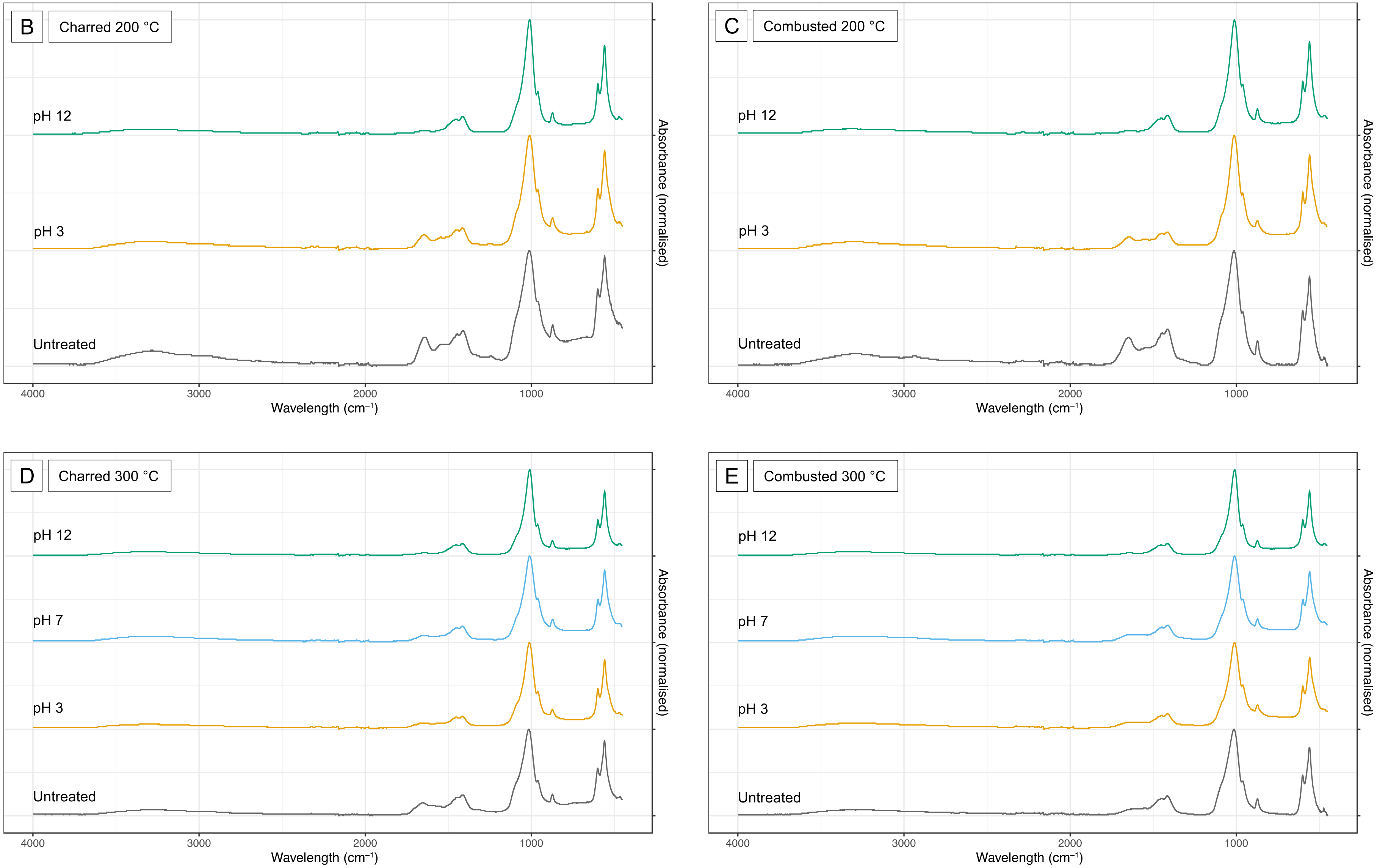
B
Charred 200 °C
pH 12
pH 3
Untreated
C
Combusted 200 °C
D
Charred 300 °C
pH 7
E
Combusted 300 °C
Absorbance (normalised)
4000
3000
2000
1000
Wavelength (cm⁻¹)

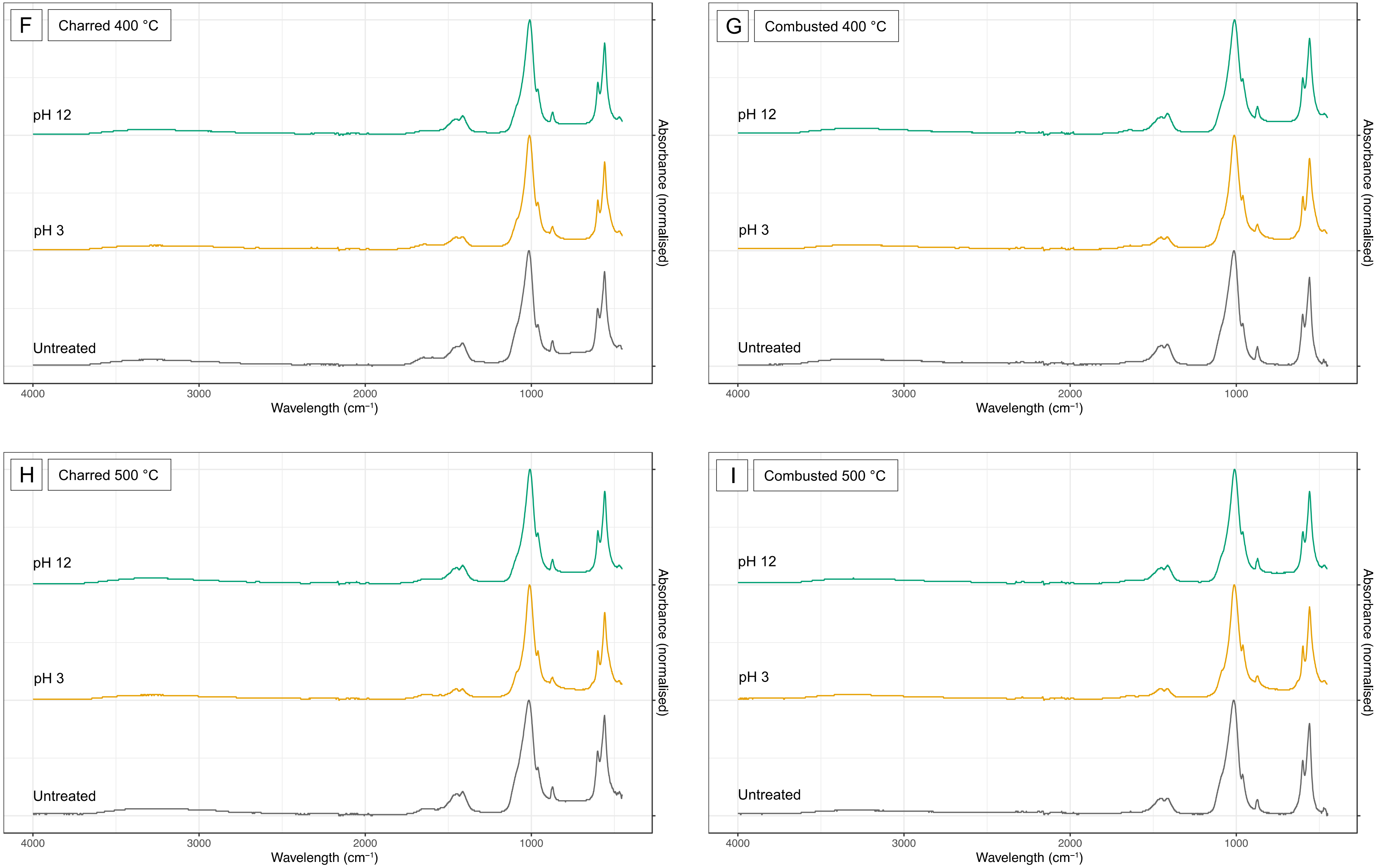
F
Charred 400 °C
pH 12
pH 3
Untreated
Absorbance (normalised)
4000
3000
2000
1000
Wavelength (cm⁻¹)
G
Combusted 400 °C
pH 12
pH 3
Untreated
Absorbance (normalised)
4000
3000
2000
1000
Wavelength (cm⁻¹)
H
Charred 500 °C
pH 12
pH 3
Untreated
Absorbance (normalised)
4000
3000
2000
1000
Wavelength (cm⁻¹)
I
Combusted 500 °C
pH 12
pH 3
Untreated
Absorbance (normalised)
4000
3000
2000
1000
Wavelength (cm⁻¹)

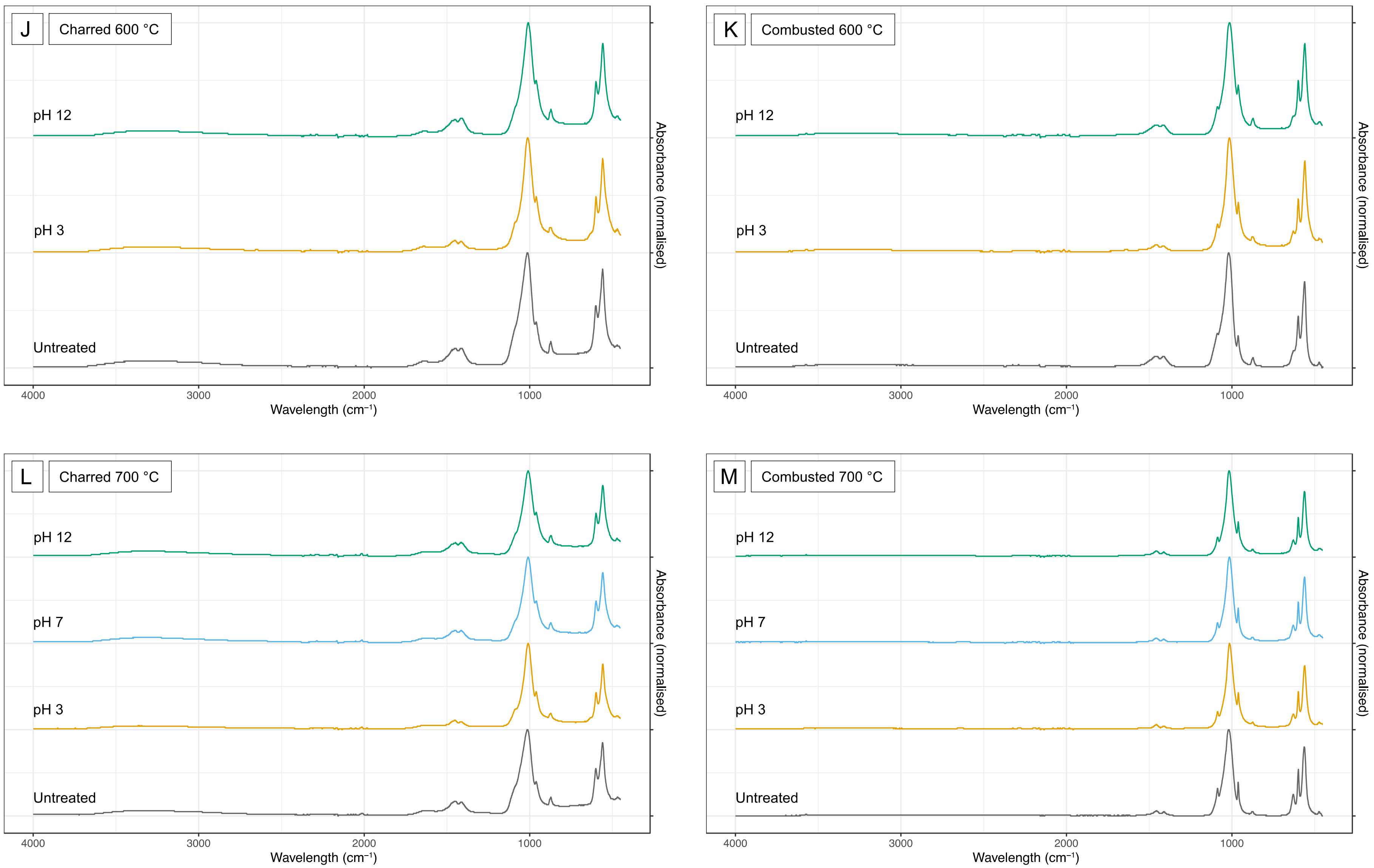
J
Charred 600 °C
pH 12
pH 3
Untreated
K
Combusted 600 °C
L
Charred 700 °C
pH 7
M
Combusted 700 °C
Absorbance (normalised)
Wavelength (cm⁻¹)
4000
3000
2000
1000

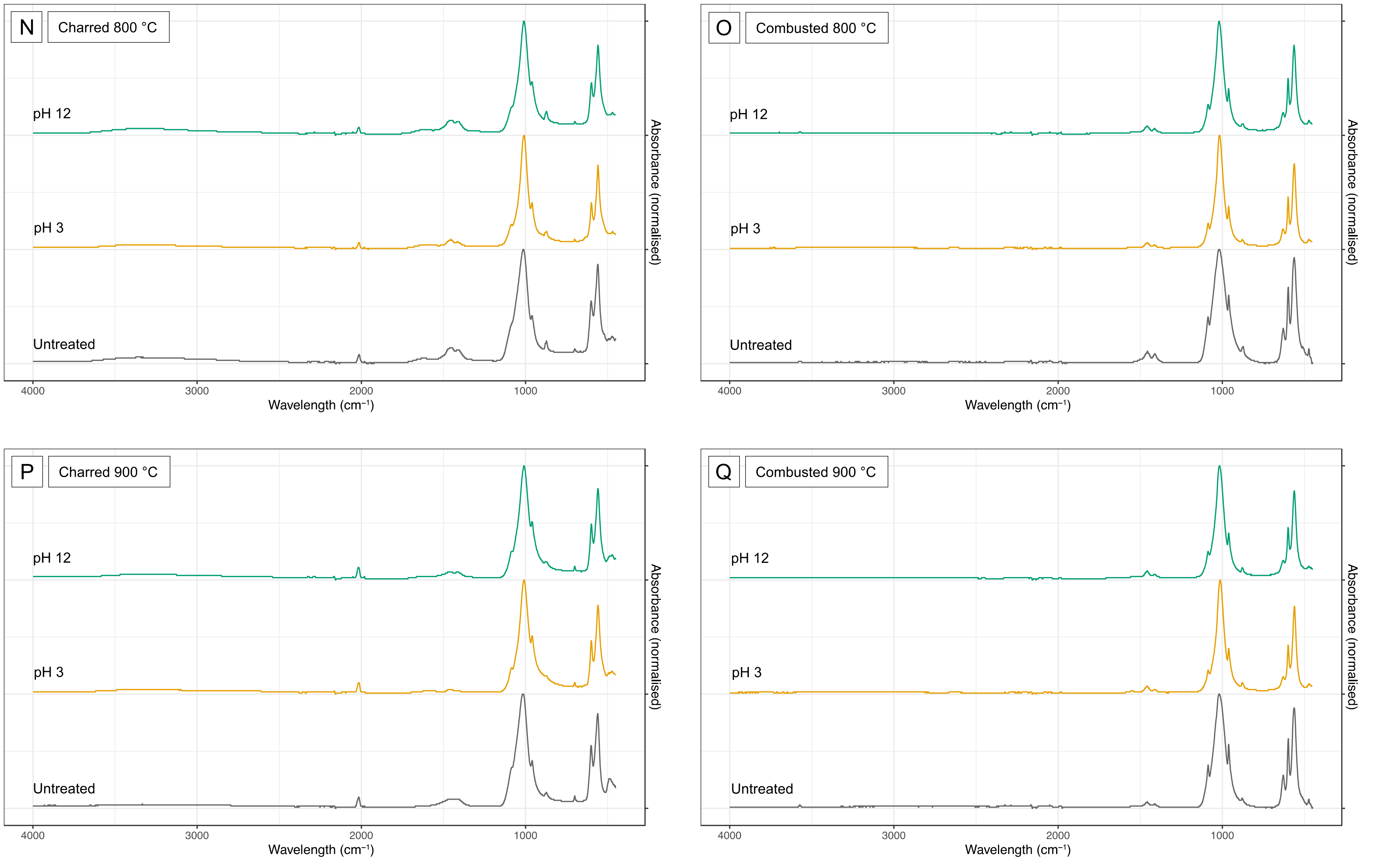
N
Charred 800 °C
pH 12
pH 3
Untreated
4000
3000
2000
1000
Wavelength (cm⁻¹)
Absorbance (normalised)
O
Combusted 800 °C
pH 12
pH 3
Untreated
4000
3000
2000
1000
Wavelength (cm⁻¹)
Absorbance (normalised)
P
Charred 900 °C
pH 12
pH 3
Untreated
4000
3000
2000
1000
Wavelength (cm⁻¹)
Absorbance (normalised)
Q
Combusted 900 °C
pH 12
pH 3
Untreated
4000
3000
2000
1000
Wavelength (cm⁻¹)
Absorbance (normalised)

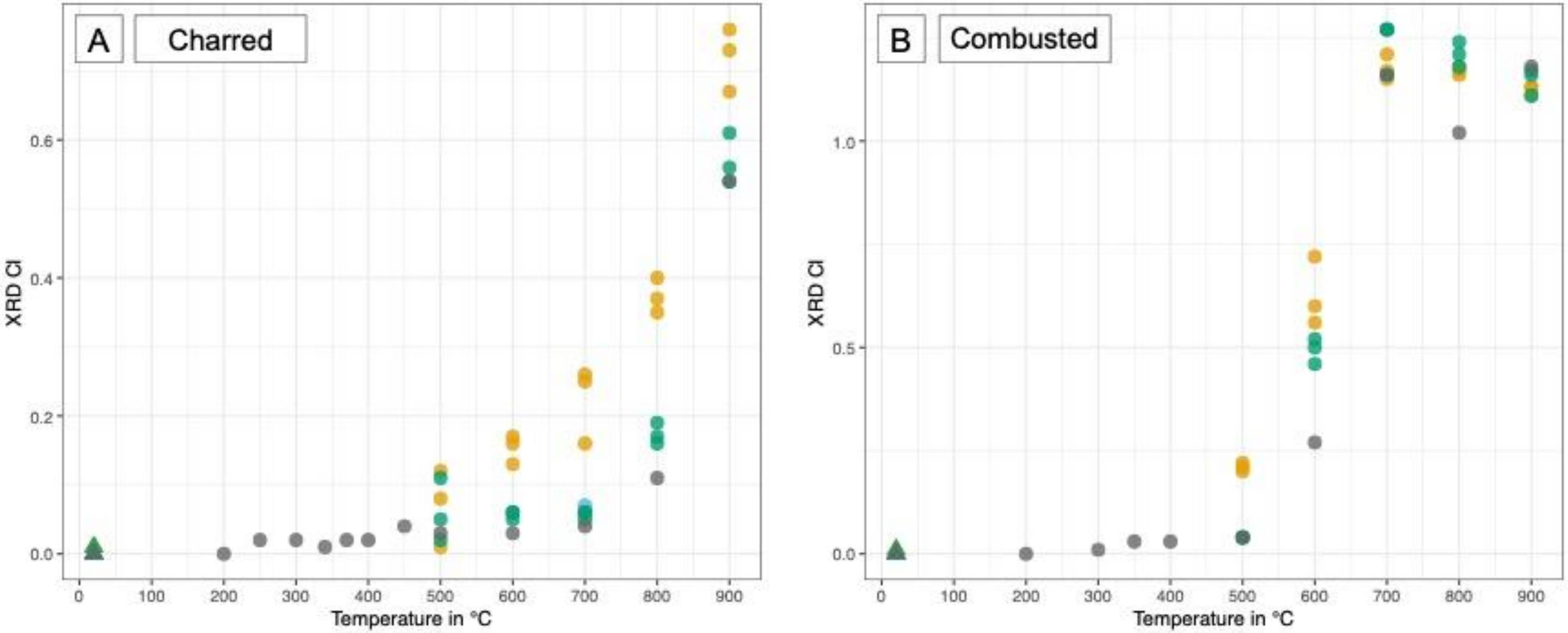

Figure SI.9: Graphs showing the variation in XRD Crystallinity Index values in charred (A) and combusted (B) bone as a result of pH exposure. Triangles = unheated bone, circles = heated bone, grey = untreated samples, yellow = pH 3 samples, blue = pH 7 samples, green = pH 12 samples.

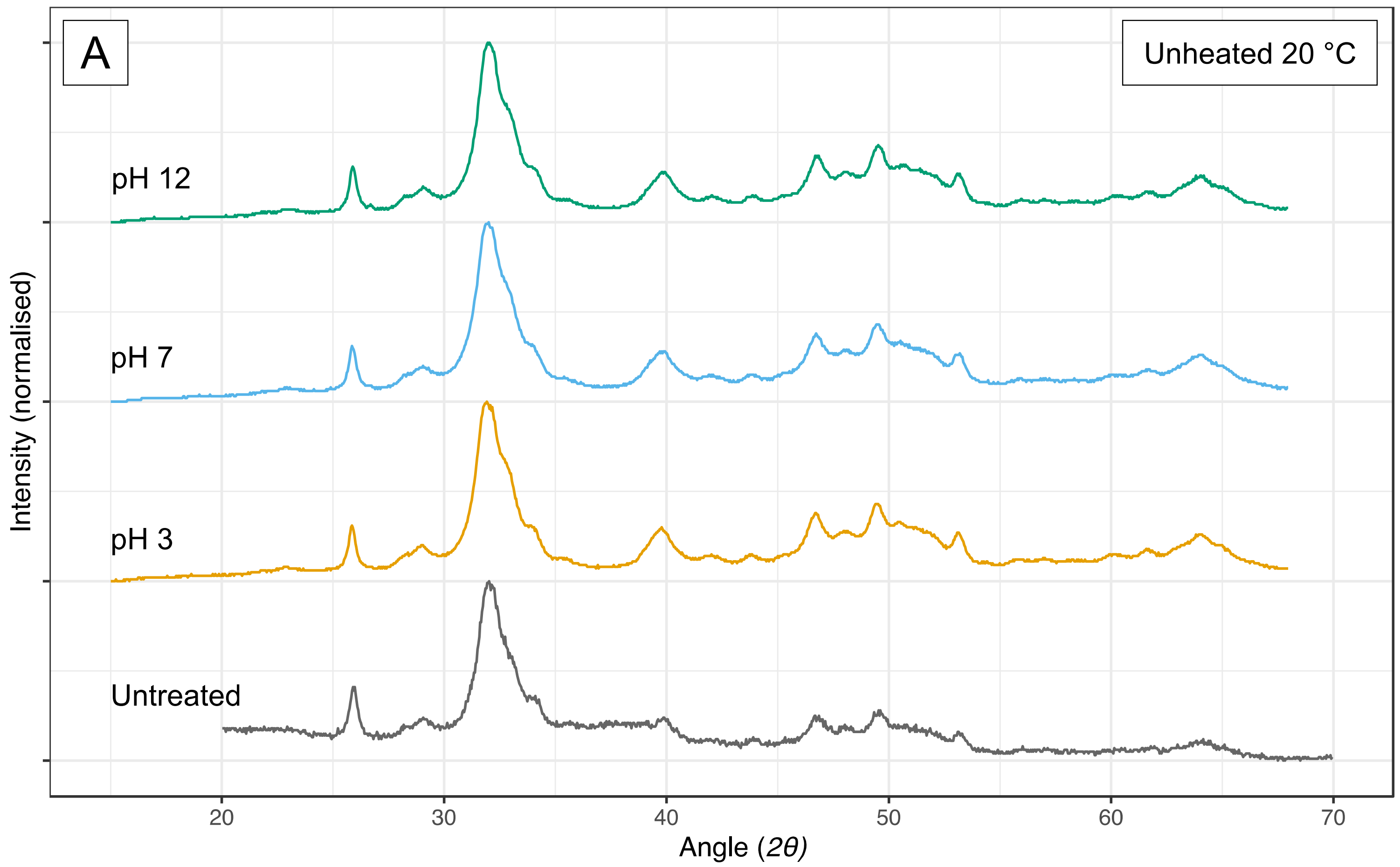

Figure SI.10a-k: XRD diffractograms for all temperature-pH combinations, compared to their untreated equivalent. Unheated (A) above, charred and combusted bone (B-K) below, with charred bone in the left column and combusted bone in the right column. Grey = untreated, yellow = pH 3, blue = pH 7, green = pH 12. For peak identifications see Fig. 3 in the main text. SI.10f and g are duplicates of Fig. 3a and b presented in the main text.

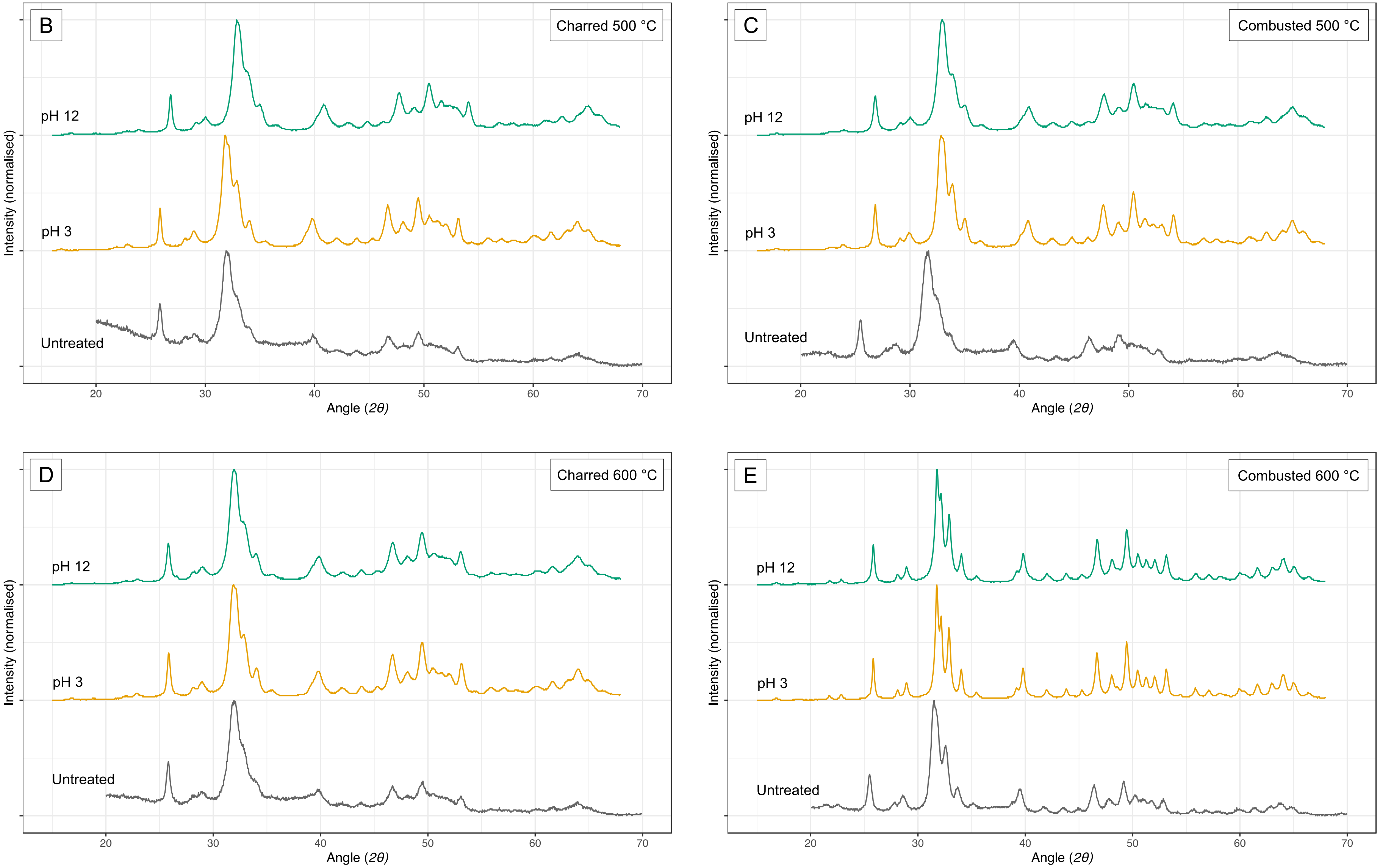
B
Charred 500 °C
C
Combusted 500 °C
D
Charred 600 °C
E
Combusted 600 °C
pH 12
pH 3
Untreated
Intensity (normalised)
Angle (2θ)
20
30
40
50
60
70

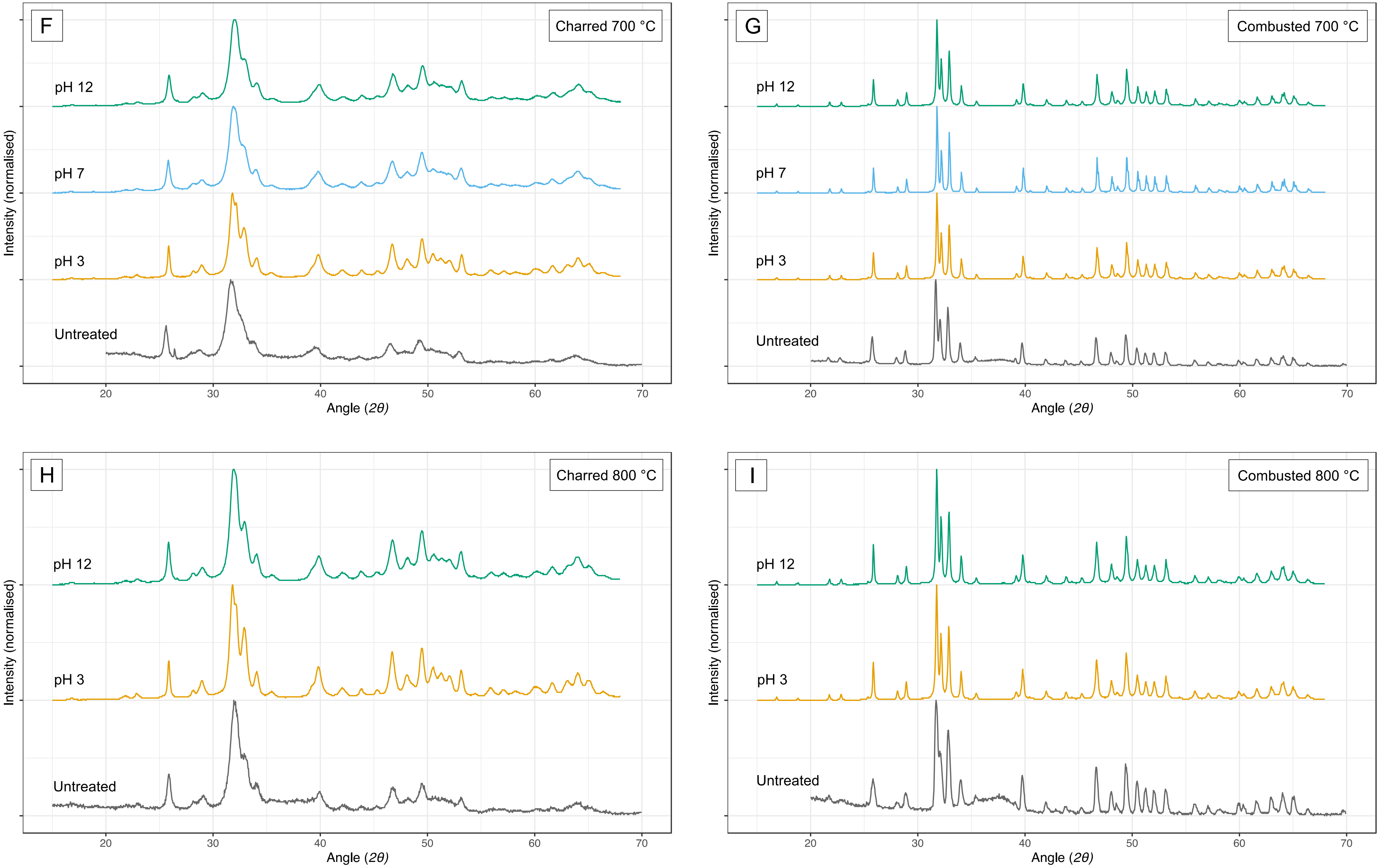
F
Charred 700 °C
G
Combusted 700 °C
H
Charred 800 °C
I
Combusted 800 °C
pH 12
pH 7
pH 3
Untreated
Intensity (normalised)
Angle (2θ)
20
30
40
50
60
70

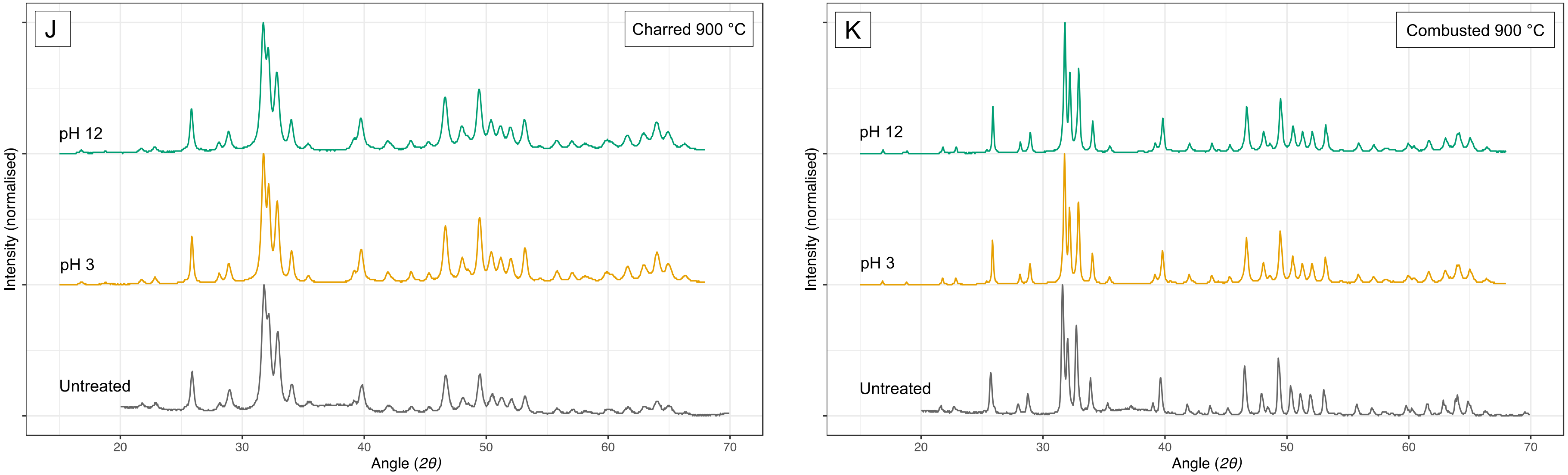
J
Charred 900 °C
pH 12
pH 3
Untreated
Intensity (normalised)
Angle (2θ)
20
30
40
50
60
70
K
Combusted 900 °C
pH 12
pH 3
Untreated
Intensity (normalised)
Angle (2θ)
20
30
40
50
60
70
